# Inflammatory, Functional, and Compositional Changes of the Uterine Immune Microenvironment in a Lymphangioleiomyomatosis Mouse Model

**DOI:** 10.33696/immunology.7.227

**Published:** 2025

**Authors:** Danielle S. Stiene, Andrew R. Osterburg, Lori B. Corsarie, Nick R. Balzarini, Mario Medvedovic, Michael T. Borchers

**Affiliations:** 1 University of Cincinnati College of Medicine, Division of Pulmonary, Critical Care and Sleep Medicine, Department of Internal Medicine, PO Box 45267-0564, Cincinnati, OH 45267, USA; 2 Department of Biostatistics, Health Informatics and Data Science, University of Cincinnati College of Medicine, Cincinnati, OH 45267-0056, USA

**Keywords:** Inflammation, NK Cells, Lymphangioleiomyomatosis, Uterus, Single-cell RNA sequencing

## Abstract

Lymphangioleiomyomatosis (LAM) is a rare, female-dominated pulmonary cystic disease. Cysts that develop in LAM are characterized by the presence of smooth muscle-like (LAMCore) cells in the periphery. These cells harbor mutations in *Tuberous Sclerosis Complex 1* or *2* (*TSC1/2*), driving uncontrolled proliferation through the mTORC1 pathway. LAMCore cells originate from an extrapulmonary source. Published data supports the uterine origin of LAMCore cells that metastasize from the uterus to precipitate pulmonary function destruction. Immune evasion is hypothesized to occur to allow seeding of the lungs from the uterus. This evasion specifically involves dysfunctional NK cells to allow aberrant proliferation and migration from the tissue. Single-cell RNA sequencing revealed changes in chemokine and cytokine protein and receptor expression in uterine NK (uNK) and other immune cell populations in a uterine-specific *Tsc2*-knockout mouse model of LAM. ELISA data revealed increased concentrations of multiple pro-inflammatory cytokines in the sera of aged *Tsc2*-knockout mice. Flow cytometry, IHC, and functional assays identified compositional and functional insufficiencies of the uNK cells in *Tsc2*-knockout mice. Furthermore, depletion of NK cells led to the increased development of pulmonary metastases. These data suggest an inflammatory feedback loop affecting multiple cell types including uNK cells, macrophages, and neutrophils. This leads to alterations in immune cell function and composition which allow for LAMCore cell metastasis from the uterine tissue, which may provide a novel mechanism for LAM development.

## Introduction

Lymphangioleiomyomatosis (LAM) is a rare, malignant, female dominated pulmonary neoplasm with symptoms typically presenting during child-bearing years. LAM is characterized by progressive cystic destruction of the pulmonary parenchyma that leads to functional decline and eventual organ failure. A distinct characteristic of LAM is the presence of nodules that can be found on the periphery of some cysts that contain transformed myometrial (LAMCore) cells. These LAMCore cells, originating from the uterus, harbor mutations in both copies of the *Tuberous Sclerosis Complex 1* or *2* (*TSC1/TSC2*) genes, most commonly *TSC2*, that are not found within other cells. This loss of function results in downstream upregulation of the mammalian target of rapamycin complex 1 (mTORC1) pathway leading to uncontrolled proliferation and survival of the LAMCore cell population [1–5]. To date, the only FDA-approved treatment for LAM is the mTORC1 inhibitor Rapamycin. While the drug can halt or slow disease progression, it is only cytostatic, and disease progression resumes when the drug is withdrawn [6,7]. Thus, there is a need for improved treatments for LAM.

Data supports the extrapulmonary origin of the *TSC2*-null LAMCore cells. These include the reoccurrence of LAM within transplanted lungs [8–11], the expression of receptors for progesterone and estrogen [12], uterine leiomyomas harboring LAMCore cells [13–16], and the presence of LAMCore cell clusters found within the circulatory system and renal angiomyolipomas [17]. Recently, single-cell RNA sequencing (scRNAseq) on matched patient lung and uterine tissues convincingly supports the uterine origin of the LAMCore cells. These data demonstrate that LAMCore cells are a transcriptomically unique population distinct from the normal resident cells within the lungs, that LAMCore cells found within each tissue share the same *TSC* mutation, and the transcriptome of the LAMCore cells are closest to that of uterine tissues [18]. From this, the uterus requires more study in the context of LAM to understand disease pathogenesis.

Throughout disease progression, immune evasion is hypothesized to allow for the continual seeding of the lungs with LAMCore cells. Natural killer (NK) cells have various functions, one being tumor immunity and surveillance. NK cell function is determined by the balance of activating and inhibitory receptors binding to ligands expressed on the surface of target cells without the need for antigen priming. In a healthy state, the expression of self-Major Histocompatibility Complexes (MHCs) and ligands for inhibitory receptors, such as CD94/NKG2A, prevent activation of NK cells and killing of healthy cells [19,20]. Conversely, binding of activating receptors, such as NKG2D, on the NK cell by stress-induced ligands on the target cell leads to NK cell activation [21–24]. Release of cytotoxic granules containing perforins and granzymes by the NK cell induces apoptosis pathways in the target cell. NK cells are also activated by cytokines, such as interleukin-12 (IL-12), to release pro-inflammatory cytokines such as interferon gamma (IFNγ). This activates other proximal immune cells, such as dendritic cells (DCs), macrophages, and other lymphocytes [22,25–29]. Dysregulation of these NK cell functions can lead to altered tumor immunity [30–32]. Thus, the NK cell function in tumor environments is important for proper tumor cell identification and elimination.

Similar to other immune cell populations, NK cells have tissue-specific phenotypes and functions. NK cells express established markers, such as CD56 and CD16 in humans and NK1.1 and NKp46 in mice but lack expression for other lineage markers such as CD3. While NK cells express these receptors, other receptor expression varies in a tissue specific manner to control activity [33–35]. For example, pulmonary NK cells express markers to influence function towards cytotoxicity over cytokine production [34]. In contrast, uterine NK (uNK) cells express a combination of markers focused on cytokine production over cytotoxicity [36–38]. A majority of research focuses on decidual NK (dNK) cells during pregnancy, while studies examining the function and composition of uNK cells in a virgin or non-pregnant uterus are less abundant. Data suggests that uNK cells present in both the non-pregnant tissue and decidua originate from a heterogenous population of tissue-resident [39,40] and circulating NK cells that have migrated into the tissue [41–43]. These uNK cells express a unique combination of surface receptors that contribute to the function in the uterus and prepare the tissue for implantation [36,44–46]. In pathological conditions such as endometriosis or recurrent pregnancy loss, alterations in uNK cell number, receptor expression, and function can contribute to disease [47–55]. Therefore, the uNK cells in LAM patients may be implicated in the pathogenesis of disease.

Little is known about immune system alterations or dysfunction in LAM patients. Our lab has previously published data regarding changes of the NKG2D receptor-ligand axis in the lungs and peripheral blood of LAM patients [56]. While the data suggests an altered NK cell phenotype, the tissues tested are from patients who are in the middle or end stages of disease. Thus, the NK cell phenotype at the beginning of disease may be different compared to the phenotype of NK cells later in the disease. Despite convincing evidence supporting the uterine origin of LAMCore cells, the uterus remains understudied within the context of LAM, attributed to a delay in diagnosis [1–4]. To circumvent this issue, we have turned to an established uterine-specific *Tsc2*-knockout mouse model for LAM. This model restricts the loss of *Tsc2* (*Tsc2^fl/fl^*) to the uterus by using Cre recombinase expression driven by the progesterone receptor promotor (*PR^Cre^*). Both copies of *Tsc2* are deleted (*Tsc2^fl/fl^PR^Cre^*) selectively in the uterus while maintaining immunocompetency (*Tsc2*-knockout mice). This provides the ability to study the immune system during disease pathogenesis, which is relatively understudied in patients. Previously published data using this model shows a marked uterine hyperproliferation of both endometrial and myometrial layers, indicating uncontrolled cell proliferation due to mTORC1 upregulation. Although patients do not see considerable uterine manifestations during disease, this model reflects the gene knockout that occurs in the population of transformed myometrial cells in patients. A caveat of this model is the inability to age for cystic pulmonary development due to uterine hypertrophy, a focus for future studies. Aged mice develop a pulmonary phenotype characterized by the presence of lung nodules containing cells that express LAMCore cell markers consistent with those observed in human disease. These include phospho-S6 ribosomal protein (pS6), alpha smooth muscle actin (αSMA), estrogen receptor alpha, and the progesterone receptor [57]. Therefore, this mouse serves as a model for the initiation and progression of disease.

We aimed to characterize the uNK cell populations using this model, as data lacks regarding the composition and function of these cells in the context of LAM in the initial stages of disease. Single-cell RNA sequencing of *Tsc2*-knockout uteri reveals changes within the composition of tissue-resident immune cells, specifically macrophages, neutrophils, granulocytes, and uNK cells, and the expression of cytokines, chemokines, and the respective receptors. ELISA data from sera of *Tsc2*-knockout mice demonstrates an increase in multiple pro-inflammatory cytokines known to affect immune cells and structural cells of the uterus. We continued our studies focusing on the uNK cells in this mouse model, as data regarding these tissue-resident cells is unstudied in animal models and LAM patients. We hypothesized that uNK cells were hyporesponsive within the uterus, allowing for LAMCore cell proliferation and migration to the lungs. We observed changes in overall uNK cell numbers within the tissue, increases in uNK cells expressing the inhibitory receptor NKG2A, and baseline IFNγ production indicating changes in uNK cell function. Depletion of NK cells revealed a functional need for the NK cell populations, as evidenced by increased pulmonary nodule formation. Therefore, the uterine inflammatory environment not only drives uNK cell dysfunction, but also infiltration and activation of additional immune cells to allow LAMCore cell metastasis to the lung.

## Methods

### Animals

Experimental mice were generated by crossing *Tsc2^fl/fl^* mice with those expressing Cre recombinase driven by the PR promoter (*PR^Cre^*), gifted from the University of Rochester [57]. Female mice expressing the uterine-specific *Tsc2*-knockout (*Tsc2^fl/fl^PR^Cre^*) and littermate controls (*Tsc2*^fl/fl^PR^+/+^) were used from 6-32 weeks of age, depending on experimental need. Age matched C57BL/6J mice were purchased from the Jackson Laboratory (Bar Harbor, ME) as needed for experiments. All mice were housed in the University of Cincinnati animal care facilities. Experimental procedures were performed in accordance with the Institutional Animal Care and Use Committee at the University of Cincinnati Medical Center. Only female mice were utilized, as this is a female-prevalent disease and genotype is restricted to a female-specific organ. For all studies, a minimum of 3 control and 3 *Tsc2*-knockout female mice were used. There was no randomization and investigators were not blinded.

### Uterine tissue digestion and lymphocyte isolation

Whole uteri were weighed prior to experimental use. Uteri removed from control mice were digested as a whole. Ovaries and the lower Female Reproductive Tract (FRT) structures were not included in tissue processing. No more than 1g of a *Tsc2*-knockout uterus was digested, and the remaining tissue was formalin-fixed for immunohistochemistry (IHC). Digestion to isolate immune cells was adapted from Rodriguez-Garcia, *et al.* [58]. The uterine tissue was minced using scissors in 1X HBSS (Gibco) modified with 100 U/mL penicillin-streptomycin (ThermoFisher), 0.35 g/L NaHCO_3_ and 20 mM HEPES (Gibco) in a tissue culture plate on ice to prevent tissue drying. After mincing, the tissue was digested for 45 minutes at 37°C and 120 rpm in the modified HBSS with Liberase TL (SigmaMillipore/Roche) at 75 uL/100 mg tissue and 0.01% DNase (ThermoFisher) at a total volume of 5 mL/g tissue. The tissue was transferred onto a 70 μm filter pre-wetted with 2 mL of normal HBSS. The digestion well was rinsed with 3 mL HBSS to collect cells and washed over the tissue on the filter. The plunger of a 5 mL syringe was used to gently break up the tissue on the filter. The tissue was rinsed with 10 mL HBSS, and the cell suspension was centrifuged at 400 g for 10 minutes, at 4°C, and resuspended in 3-5 mL of RPMI (Gibco), depending on the amount of tissue digested. Cells were slowly layered on top of an equal volume of 70% Percoll for density-gradient lymphocyte isolation and centrifuged at 400 g for 25 minutes with no brakes at room temperature. The cell layer was removed, washed with RPMI, centrifuged at 400 g for 10 minutes, and RBC lysis (Biolegend) performed if necessary. The cells were resuspended in RPMI, filtered over a 70 μm filter, and cell number and viability calculated via Trypan blue (Gibco) by hemocytometer. If cell death was greater than 20%, dead cell removal was performed according to the manufacturer’s protocol (Militenyi, Auburn, CA) prior to downstream applications. Spleens were mechanically digested by breaking up the tissue between frosted slides in 10 mL of PBS on ice. The splenic single-cell suspension was then processed alongside the uterine cells from density gradient.

### Fixed flex single cell RNA sequencing analysis

Mice expressing the uterine-specific *Tsc2*-knockout and age matched C57BL/6J (Jackson Labs, Bar Harbor, ME) were aged to 24 weeks old. Mice were euthanized, the uteri removed, and the tissue sat on ice in PBS until processing. The tissues were fixed, digested, and a single-cell suspension processed for long-term storage according to the manufacturer’s protocol (Demonstrated protocol CG000553, 10X Genomics). Cells were processed, RNA isolated, and sequenced via the Cincinnati Children’s Single Cell Genomics Facility (RRID: SCR_022653) and the Cincinnati Children’s Genomics Sequencing Facility (RRDI: SCR_022630).

The raw data was de-multiplexed, aligned to the Mus musculus (mm10) reference genome, and quantified using the Cell Ranger [59] processing pipelines. Further quality control filtering, normalization, integration and clustering was performed using the *Seurat* package (version 5) [60]. Cells (SC) with low count depth, few detected genes and high fraction of mitochondrial gene counts were removed. The data was normalized using regularized negative binomial regression as implemented in the latest version (version 2) of *sctransform* [61], integrated using the reciprocal PCA integration [62], clustered using Louvain-Jaccard graph community detection algorithm [63,64] and visualized in two dimensions by UMAP [65] dimensionality reduction. Differential expression analysis between *Tsc2*-knockout and control mice for each cluster separately was performed using *pseudo-bulk* approach [66]. Regularized counts were aggregated for each cluster and each mouse and the differential gene expression analysis statistical analysis using the negative binomial model as implemented in *edgeR* package [67].

Further pathway analyses were investigated using the Reactome database (Analysis Tools, V92, https://reactome.org/) on differentially expressed genes (DEGs) in the *Tsc2*-knockout mice compared to controls in each cluster. Briefly, DEGs were put into the Reactome Analysis Tool, converted to human orthologs, and statistical analysis performed on pathways analyzed for gene enrichments. Any pathways with an FDR value <0.05 were noted [68,69].

### Flow cytometry

Cells were resuspended in flow buffer (FB) (1× Ca/Mg-free PBS, 0.5% BSA, 0.1% sodium azide) and stained with a fixable viability dye (1:1000 dilution, Biolegend) at 4°C for 20 minutes in the dark. Cells were washed, centrifuged at 400 g for 10 minutes, and resuspended at a concentration of 10^6^ cells/100 uL FB. Cells were blocked using purified mouse anti-CD16/32 (isotype Rat IgG2a, λ, Biolegend) for 15 minutes at room temperature in the dark. After blocking, cells were surface stained for 30 minutes at 4°C with the following antibodies: anti-CD45 (clones I3/2.3 or 30-F11, Biolegend), anti-CD3 (clone 17A2, Biolegend), anti-NK1.1 (clone PK136, Biolegend), anti-NKp46 (clone 29A1.4, Biolegend), anti-CD49b (clone DX5, Biolegend), anti-NKG2D (clone CX5, Biolegend), and anti-NKG2A (clone 16A11, Biolegend) in various combinations dependent on experimental needs. All antibodies were titrated prior to use for optimized staining concentrations. Cells were washed, resuspended, and fixed in 1% paraformaldehyde (PFA). Cells were analyzed on an Attune 2-laser (488 nm, 633 nm), 6-detector cytometer. FCS files were analyzed using FCSExpress (DeNovo Software, v7). Compensation matrices were determined using UltraComp eBeads (eBiosciences, ThermoFisher), and appropriate fluorescence minus one controls used. Cells were first gated on lymphocytes by comparing Forward Scatter Area vs Side Scatter Area, viable cells discriminated in the negatively stained population, and doublets excluded by gating on single cells (FSC-H vs FSC-A). CD45^+^CD3^−^ cells were gated, and NK cells defined by NK1.1^+^NKp46^+^ dual positivity. These NK cells were then analyzed for the presence of additional NK cell markers. Standardization of NK cells was performed by taking the number of NK cell events and dividing by the total milligrams of whole uteri for controls. For *Tsc2*-knockout mice, NK cell events were divided by the weight of digested tissue to determine the ratio of cells to tissue. The cell to tissue ratio was then multiplied by total uterine weight to find the total number of NK cells.

### Intracellular staining for flow cytometry

Uterine and splenic lymphocytes were isolated as described above and rested overnight at 37°C/5% CO_2_ with IL-2 (40U/ mL, Peprotech) and IL-15 (10ng/mL, Peprotech) in the following media: IMDM with phenol red, 10% FCS (Gibco), 2 mM L-glutamine (Gibco), 100 U/mL penicillin-streptomycin (Gibco), 0.1 mM NEAA (Gibco), 50 uM βME. Following rest, cells were treated with the following treatment conditions for 4 hours at 37°C/5% CO_2_: IL-2 (40 U/mL) + IL-15 (10 ng/mL), IL-2/IL-15 + LPS (0.2 mg/mL) (ThermoFisher), IL-2/IL-15 + Cell Stimulation Cocktail (0.2 mg/mL) (Invitrogen), or IL-2/IL-15/ and IL-12 + IL-18 (50 ng/mL both, Peprotech). Brefeldin A (Invitrogen) is added to the culture to prevent cell content release. The cells were washed in cell media and stained with a fixable viability dye (1:1000, Biolegend) for 20min at 4°C in the dark. Cells were surface stained with the following antibodies: anti-CD8 (clone 53-6.7, Invitrogen), anti-CD3 (clone 17A2, Biolegend), anti-CD4 (clone GK1.5, Biolegend), and anti-NKp46 (clone 29A1.4, Biolegend). Cells are washed and fixed in 1% PFA for 20 mins at room temperature in the dark. Cells were then washed and permeabilized twice using a 1X permeabilization buffer (Biolegend). Non-specific antibody binding was blocked using a purified anti-mouse CD16/32 (isotype Rat IgG2a, λ, Biolegend) antibody for 15 minutes at room temperature in the dark. Cells were then stained for IFNγ (clone XMG1.2, Biolegend) for 30min in the dark at 4°C. Cells were washed twice in 1X permeabilization buffer and then resuspended in FB prior to running cells on an Attune 2-laser (488 nm, 633 nm), 6-detector cytometer. CD3 staining distinguished T cells vs NK cells: CD3^+^ are T cells and CD3^−^ cells gated upon to further define NK cells by NK1.1^+^NKp46^+^ dual staining. CD4 vs CD8 staining on the CD3^+^ population was used to look at specific T cell populations. Once T and NK cell populations were defined, IFNγ staining was investigated.

### Apoptosis assays

Uterine and splenic lymphocytes were isolated as described above without performing dead cell removal. Cells were blocked using purified mouse anti-CD16/32 (isotype Rat IgG2a, λ, Biolegend) for 15 minutes at room temperature. Cells were surface stained for flow cytometry analysis using the following antibodies: anti-NK1.1 (clone PK136, Biolegend), anti-NKp46 (clone 29A1.4, Biolegend), and anti-CD3 (clone 17A2, Biolegend). Caspase-3/7 apoptosis staining was performed using the CellEvent^™^ Caspase-3/7 Green Flow Cytometry Assay Kit protocol (ThermoFisher). Cells were analyzed immediately on an Attune 2-laser (488 nm, 633 nm), 6-detector cytometer. FCS files were analyzed using FCSExpress (DeNovo Software, v7). Compensation matrices were determined using UltraComp eBeads (eBiosciences, ThermoFisher Scientific), and appropriate fluorescence minus one controls used. Graphing and statistics were performed using GraphPad Prism (v9/10). For gating, first lymphocytes (FSCA vs SSCA) were gated to isolate the single cells (FSCA vs FSCH). T cells were denoted by CD3^+^ and NK cells by CD3^−^NK1.1^+^NKp46^+^ staining on the single cells. Apoptosis was then assessed by comparing Caspase-3/7 vs 7-AAD staining.

### Immunohistochemistry

After 24 hours in 10% formalin, uterine or lung tissues were dehydrated in an ethanol graded series. Tissues were processed and embedded in paraffin at the Integrated Pathology Research Facility at Cincinnati Children’s Hospital Medical Center (RRID # SCR_022637) or in-house. Two non-sequential sections were stained with H&E to visualize pathological changes that may have occurred in the tissues. Additional non-sequential sections were stained via immunohistochemistry against the following murine targets: anti-NCR1 (1:500, Abcam ab233558) or anti-pS6 (1:500, Cell Signaling Technology #4858).

Five micrometer sections from FFPE tissues were deparaffinized and rehydrated using a graded alcohol series. Heat-mediated antigen retrieval was performed according to antibody specifications by the manufacturer. Endogenous peroxidase activity was blocked using BloxAll (Vector Laboratories, Inc) or hydrogen peroxide. Non-specific antibody binding was blocked via serum prior to primary antibody incubation overnight at 4°C. Slides were incubated with the corresponding secondary antibody at 1:500-750 dilution, dependent on staining optimization, for 30 minutes at room temperature. HRP (Vector Laboratories, Inc.) was applied and incubated for 30 minutes at room temperature. Slides were developed using DAB/Peroxidase (Vector Laboratories, Inc.), counterstained with hematoxylin, dehydrated, and coverslips fixed. Multiple images were taken across the whole section for accurate representation depending on tissue structure. Images were quantified using Fiji (ImageJ, NIH) and data averaged across all images for one value per sample. Graphing and statistics were performed using GraphPad Prism (v9/10). In figures, all images were increased by one level in overall brightness to adjust for low exposure when imaging.

### LEGENDPlex ELISA assay

Blood was collected via cardiac puncture and placed into a Mini Collect Serum Sep tube (Greiner Bio-One). Samples were centrifuged for 1500 g for 10 minutes, serum layer transferred into a 0.6 mL Eppendorf tube and stored at −80°C until use. Samples were thawed on ice immediately prior to use. The LEGENDPlex Mouse Inflammation Panel (13-plex) (Biolegend) was performed following the manufacturer’s protocol. Samples were run on an Attune 2-laser (488 nm, 633 nm), 6-detector cytometer and analyzed using the LEGENDPlex data analysis software (Biolegend). Graphing and statistics were performed using GraphPad Prism (v9/10).

### *In vivo* NK cell depletion studies

Mice expressing the uterine-specific *Tsc2*-knockout and littermate controls were aged 12 weeks. Mice were injected intraperitoneally (I.P.) once/week with 25 μg of an anti-NK1.1 monoclonal antibody (clone PK136) or isotype control IgG2a produced by Bio-X-Cell (West Lebanon, NH). Animals were weighed weekly throughout the study. After 12 weeks, mice were euthanized, uteri were removed, weighed, digested, and fixed as described above. Cardiac puncture was performed to collect PBMCs to analyze the effectiveness of NK cell depletion. Both uterine and blood lymphocytes were processed and stained with the flow cytometry panel described previously, exchanging the NK1.1 marker for CD49b (clone DX5, Biolegend). Lungs were perfused with warmed PBS with 0.6 mM EDTA through the left ventricle of the heart to clear blood from the lungs. Inflation-fixation with formalin was performed at a height of 15 cm. Lungs were removed, dehydrated in graded ethanol 24-hours after fixation, and processed for anti-pS6 IHC-P analysis.

## Results

### Single-cell RNA sequencing reveals compositional and gene expression changes in immune cell populations

**Figure 1.**
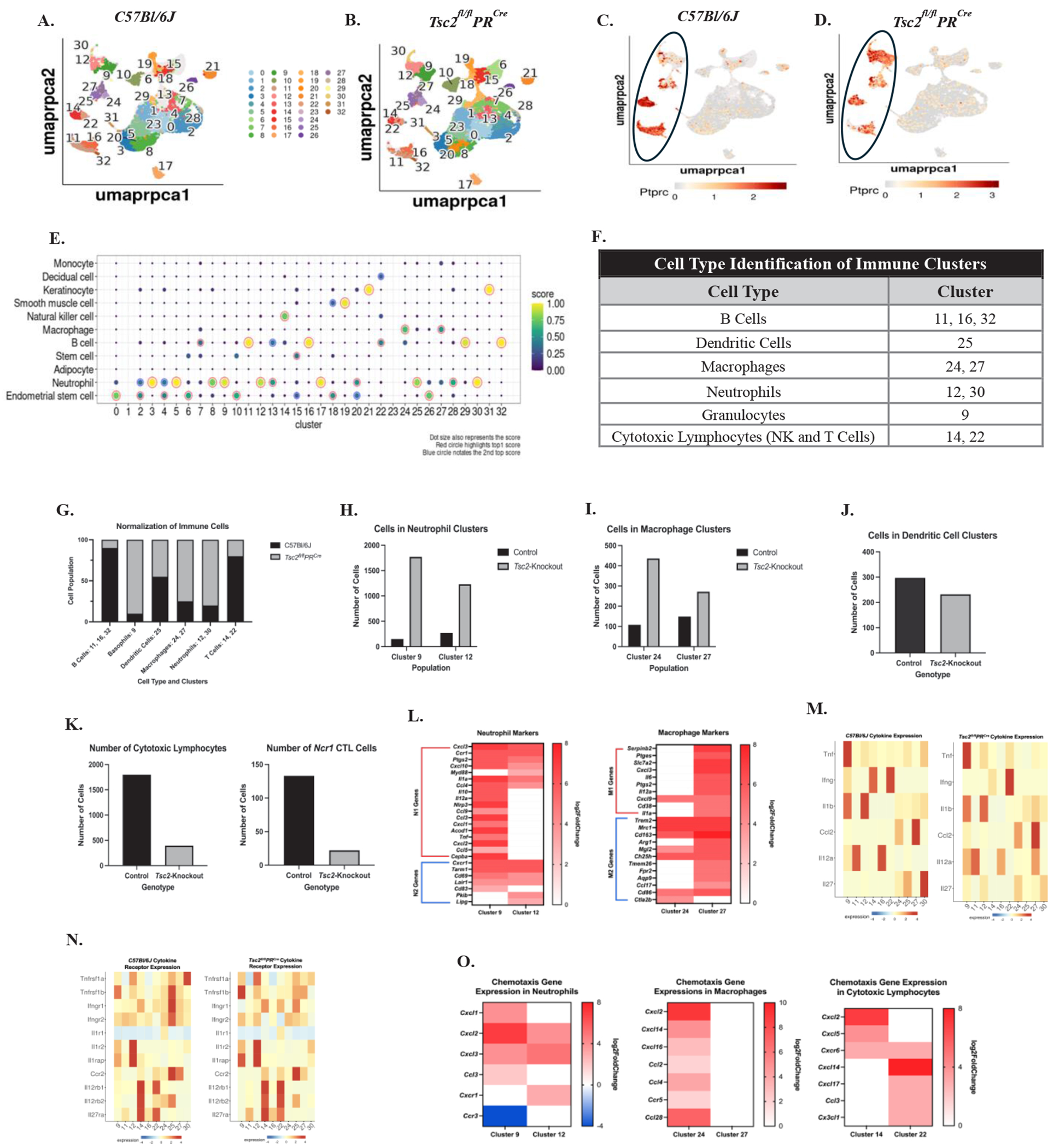
Single-cell RNA Sequencing reveals differences in immune cell composition between control and *Tsc2*-knockout mice. Five Control and five *Tsc2*-knockout tissues were harvested, fixed, and single-cell suspensions generated for single-cell RNA sequencing using the 10X Genomics Chromium Next GEM Single Cell Fixed RNA Profiling Platform. **A**. and **B.** show 32 identified cell clusters within the UMAP of C57Bl/6J (control) and *Tsc2*-knockout mice. *Ptprc* was used to identify the clusters where immune cells reside in control (**C.**) and *Tsc2*-knockout (**D.**) mice. The black circle denotes the immune cell clusters in each genotype. **E.** Analyses revealed NK cells reside within Cluster 14, which are grouped into the “T Cell” label on the UMAP. Two neutrophil clusters (12 and 30), a granulocytes cluster (9), two macrophage clusters (24 and 27), and three B cell clusters (11, 16, and 32) were identified as well. **F.** A table summarizing the immune cell types with the associated cluster number. **G.** The composition of labeled immune cells was normalized to 100% and control and *Tsc2*-knockout mice demonstrated varying degrees of immune cell enrichments **H.** The total number of events between control and *Tsc2-*knockout uteri in the granulocytes and neutrophil populations. **I.** The total number of events between macrophage clusters in control and *Tsc2*-knockout uteri. **J.** The total number of dendritic cell events between genotypes. **K.** The number of events in both cytotoxic lymphocyte (CTL) clusters 14 and 22 in both genotypes. To identify the presence of NK cells, *Ncr1* was used as a cell marker. **L.** Gene markers for N1 and N2 neutrophils and M1 and M2 macrophages are shown. Additional cytokine (**M.**) and cytokine receptors (**N.**) in the macrophage, neutrophil, granulocyte, and CTL populations. **O.** Differences in gene expressions for chemokines and chemokine receptors were noted in the macrophage, neutrophil, granulocyte, and CTL populations in the *Tsc2*-knockout uteri. Graphs show log2fold changes, and all genes shown are significantly differentially expressed (padj≤0.05) in the *Tsc2*-knockout uterus compared to the controls. Shades of red indicate upregulation, and blue denotes downregulation in heatmaps.

We performed scRNAseq analyses to investigate the uterine immune environment and potential transcriptomic changes to tissue-resident cells. Control and *Tsc2*-knockout uteri were collected, fixed, and single cells isolated for analyses. Thirty two individual cell clusters are present in both the control (Figure 1A) and *Tsc2*-knockout (Figure 1B) uteri. Using the CD45 gene *Ptprc* to identify the immune cells, the following clusters are immune populations: neutrophils (30 and 12), basophils (9), macrophages (24, 27), dendritic cells (25), cytotoxic lymphocytes (14, 22), and B cells (11, 16, and 32) (Figures 1C and 1D, circled). Using established cell markers [70], two distinct clusters of neutrophils were identified as clusters 12 and 30. Cluster 9 was labeled as basophils when our dataset was compared to other published sets (Figure 1G). Publications on the mouse uterine immune populations do not report basophils within the tissues [71,72]. Upon further examination, cluster 9 basophils express multiple established neutrophil markers (Figure S1). Therefore, this cluster was re-designated as a population of granulocytes to be analyzed alongside the neutrophils (Figures 1E and 1F). Cluster 30 neutrophils were negligible in number, and therefore not included in downstream analyses. Based on published data [73], cluster 9 granulocytes express N1 gene markers (pro-inflammatory neutrophils) and cluster 12 neutrophils favor N2 (anti-inflammatory neutrophils) gene expression indicating a mixture of neutrophil and other granulocyte-like populations in the uteri (Figure 1L). Two distinct macrophage clusters were identified as clusters 24 and 27. Further analyses suggest cluster 24 is comprised of more M2 (anti-inflammatory) macrophages while cluster 27 is a mixture of M1 (pro-inflammatory) and M2 macrophages (Figure 1L) [74]. B cells were identified in three distinct clusters (11, 16, and 32) (Figures 1E, 1F, and S1). Cluster 25 is a distinct dendritic cell (DC) cluster with no difference in cell numbers (Figures 1E, 1J, and S1). Multiple genes are upregulated in the *Tsc2*-knockout mice in each cluster (Figure S2). Therefore, broad transcriptional changes are observed between control and *Tsc2*-knockout mice.

In the control uteri, a high-level analysis of overall composition of immune cells showed slight enrichment in the cluster 12 neutrophils and cluster 27 macrophage populations compared to the other macrophage and granulocyte populations (Figures 1H and 1I). In contrast, the *Tsc2*-knockout uteri showed more cell numbers in the cluster 9 granulocytes and cluster 24 macrophage populations compared to the other neutrophil and macrophage clusters (Figure 1H and 1I). When normalized to 100%, enrichment of neutrophils, granulocytes, and macrophages is seen in the *Tsc2*-knockout uterus (Figure 1G). Next, we aimed to examine the uNK cell population. The total number of T and NK cells is higher within the control mice in clusters 14 and 22 (Figure 1K). Based on gene expression, uNK cells mainly reside within cluster 14 (Figure 1E). Using the gene *Ncr1*, which encodes for the NK cell-specific receptor NKp46, less NK cells are detected within the *Tsc2*-knockout uterus (Figure 1K). However, in comparison to other datasets, uNK cells are labeled as T cells—most likely cytotoxic CD8^+^ T cells or NKT cells—due to similarities in gene expression profiles (Figure 1E) [75]. The overlap of the clusters on the UMAP also demonstrates a close transcriptomic relationship between cell populations and made it difficult to single out the NK cells (Figures 1A and 1B). Therefore, these cells are referred to as cytotoxic lymphocytes (CTLs), and both clusters were further analyzed. The enrichment of macrophages, neutrophils, and granulocytes in the *Tsc2*-knockout uterus prompted further investigation into their respective cell clusters.

After identifying the immune cells, we analyzed cytokine and corresponding receptor gene expressions within the clusters. These cytokines are associated with production by specific cell types, assisting in cell identification (Figures 1M and 1N). Neutrophils and granulocytes demonstrate high expression of *Il1a* and *Il1b* in both genotypes, suggesting these cells produce these cytokines in normal and inflammatory conditions. Moderate expression of *Il1a* is seen in macrophages in *Tsc2*-knockout mice. Macrophages are the only population without decreased expression of *Il1r1* in controls, while *Il1r2* is expressed in neutrophils and granulocytes in both genotypes. *Ccr2* expression is slightly less in macrophages compared to DCs in the control mice, while expression shifts almost exclusively to DCs in the *Tsc2*-knockout mice. In the CTL populations, *Ccr2* expression favors cluster 22 in controls while expression is distributed more evenly between CTLs in the *Tsc2*-knockout mice. Macrophages express the *Ccl2* gene (MCP-1) similarly in both genotypes. *Il12a* expression becomes more diffusely expressed across clusters in the *Tsc2*-knockout mice. This suggests a role for multiple cell types to produce IL-12 in an inflammatory state. In control uteri, the expression of *Il12rb1* and *Il12rb2* is predominantly localized to CTL cluster 14, whereas in *Tsc2*-knockout uteri, expression is more uniformly distributed across both CTL clusters. *Il27ra* expression is higher in control cluster 14 CTLs compared to cluster 22 CTL expression in the *Tsc2*-knockout mice. *Ifng* is almost exclusively expressed by cluster 22 CTLs in the *Tsc2*-knockout mice while both control CTL clusters express similar levels. *Ifngr1* expression is found in DCs in both groups, although decreased in *Tsc2*-knockouts. Expression of *Ifngr2* is found more uniformly expressed across the non-CTL clusters in both genotypes. *Tnf* expression is found to be high in cluster 9 granulocytes in both genotypes of mice. The expression for the *Tnfrsf1b* gene is higher in cluster 12 neutrophils in *Tsc2*-knockout mice. Expression of the gene is high in control DCs but drops in the *Tsc2*-knockout mice. These data show that multiple immune cell populations express genes encoding cytokines and their corresponding receptors, suggesting inflammatory communication between them.

Chemoattractant genes were examined in the cluster comparisons because of high numbers of neutrophil, granulocyte, and macrophage populations in the *Tsc2*-knockout uteri (Figure 1O). In neutrophils and granulocytes, chemotactic ligands and receptor genes were increased. *Cxcl2* and *Cxcl3* were up in both populations, while cluster 9 granulocytes expressed *Cxcl1* and *Ccl3* and cluster 12 expressed *Cxcr1*. In the macrophages, all differentially expressed genes are found in cluster 24. These are *Cxcl*, *Ccl*, and *Ccr* genes. In the CTLs, *Cxcl2* and *Cxcl5* are upregulated in cluster 14 only. *Cxcr6* is differentially expressed in both clusters. The genes for *Cxcl14*, *Cxcl17*, *Ccl3*, and *Cx3cl1* are upregulated in cluster 22 CTLs only. *Ccr3* is the only gene between all neutrophils, granulocytes, macrophages, and CTLs to be downregulated (Figure 1O). These findings indicate that the expression of cytokines, chemokines, and their respective receptors is altered in response to the inflammatory environment created by hyperproliferation of the uterine tissue. Future studies will investigate the two macrophage populations to learn about their phenotype and function during disease establishment. Building on these data, downstream studies focus on the previously unstudied uNK cells to elucidate functional changes and potential roles in the initial establishment of disease.

**Figure 2.**
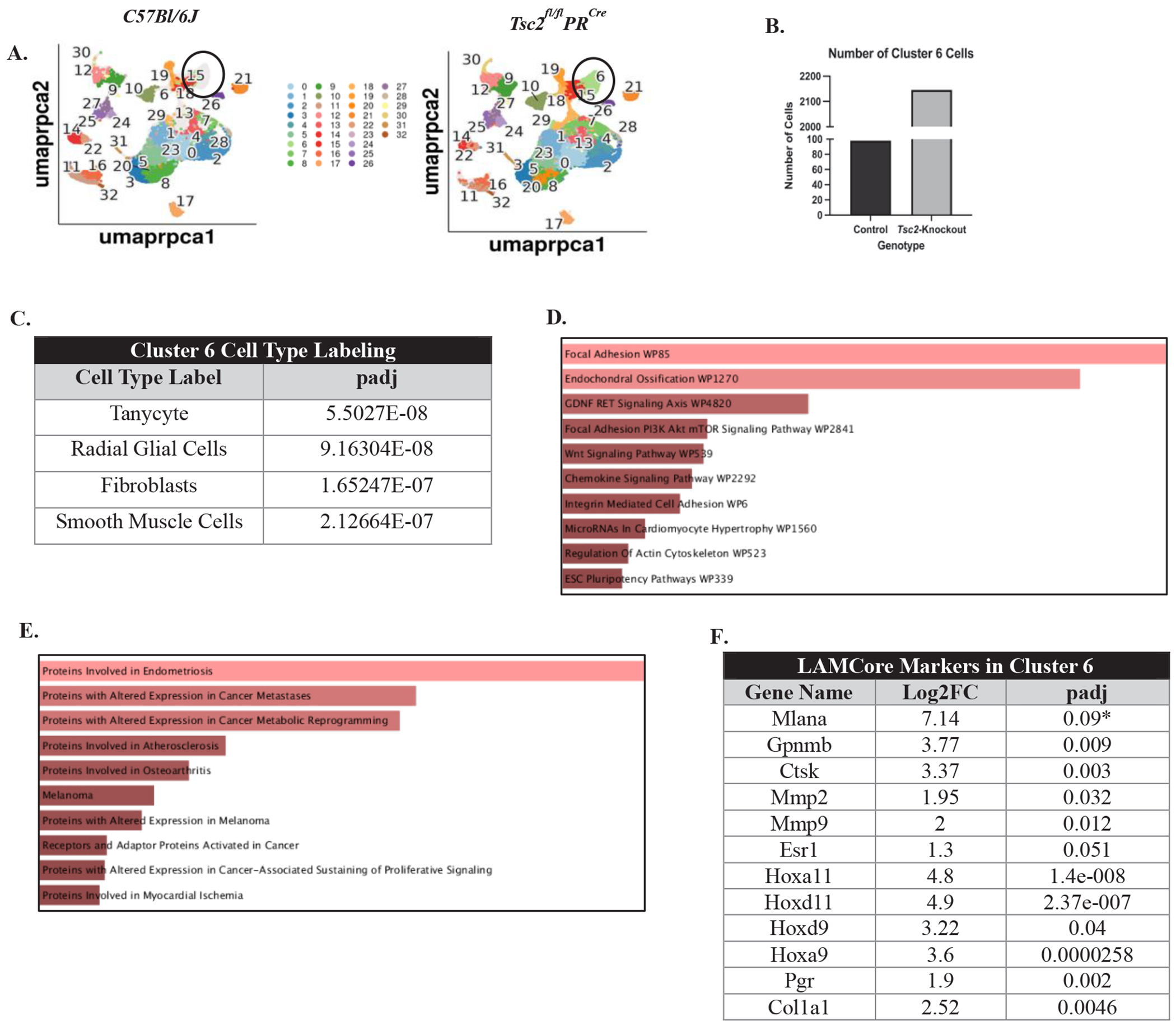
Single-cell reveals an aberrantly proliferating cell population within the *Tsc2*-knockout uteri similar to human LAM cells. Investigation into cluster 6 was performed due to the distinct lack of cells in this cluster in control mice compared to *Tsc2*-knockout mice. **A.** UMAPs of control and *Tsc2*-knockout mice with a circle denoting the location of cluster 6. **B.** Cell numbers were enriched in cluster 6 in *Tsc2*-knockout compared to controls. **C.** Cell type assignments listed the most likely labels for the cell cluster. **D.** Functional and **E.** disease pathway enrichments in the *Tsc2*-knockout mice attributed to differentially expressed genes (DEGs) in the cluster. The brighter red indicates a more significant term (see Ref. 76). **F.** Gene expression for LAMCore cell markers in cluster 6 of the *Tsc2*-knockout mice (see Ref. 18).

In addition to analyzing immune cell populations, we examined other cell clusters to determine whether they contain aberrant cells that may resemble those observed in patient samples. We found a difference between the presence of cluster 6 between control and *Tsc2*-knockout mice. Cluster 6 cells are enriched within the *Tsc2*-knockout mice (Figures 2A, circled, and 2B). When compared to other datasets, these cells are labeled as either neural-like cells, fibroblasts, or smooth muscle cells due to the DEGs within the cluster (Figure 2C). Using readily available databases [76], functions that are enriched in the DEGs from *Tsc2*-knockout mice include chemokine signaling and focal adhesion through the mTOR pathway (Figure 2D). Reactome pathway analyses of the mTORC1 pathway revealed multiple gene enrichments in the *Tsc2*-knockout cluster 6, suggesting upregulation of the pathway (Figure S4) [5,68,69]. Diseases associated with the DEGs include endometriosis, cancer metastasis, and cancer metabolism (Figure 2E). Single-cell RNA sequencing on matched LAM patient lung and uterine tissues revealed that the LAM cells, labeled as LAMCore cells, express genes unique to the population [18]. Upon comparison of these genes in our dataset, cluster 6 within the *Tsc2*-knockout mice demonstrated differential gene expression of multiple LAMCore markers (Figure 2F). Control cluster 6 expressed no LAMCore markers (Figure S3A). Expression of the well-known LAMCore markers *Gpnmb* and *Mlana* were examined in the control and *Tsc2*-knockout mice. Expression of *Gpnmb* was found in normal cells in controls, as expected. *Mlana* expression was not found in any cell type in the controls. The expression of both these genes was found in cluster 6 in the *Tsc2*-knockout mice (Figures S3B and S3C). Therefore, our mouse model recapitulates the development of a transformed myometrial cell due to the loss of *Tsc2* seen in the uteri and lungs of LAM patients.

### Immune response and tissue structure pathways are enriched in immune cells

**Figure 3.**
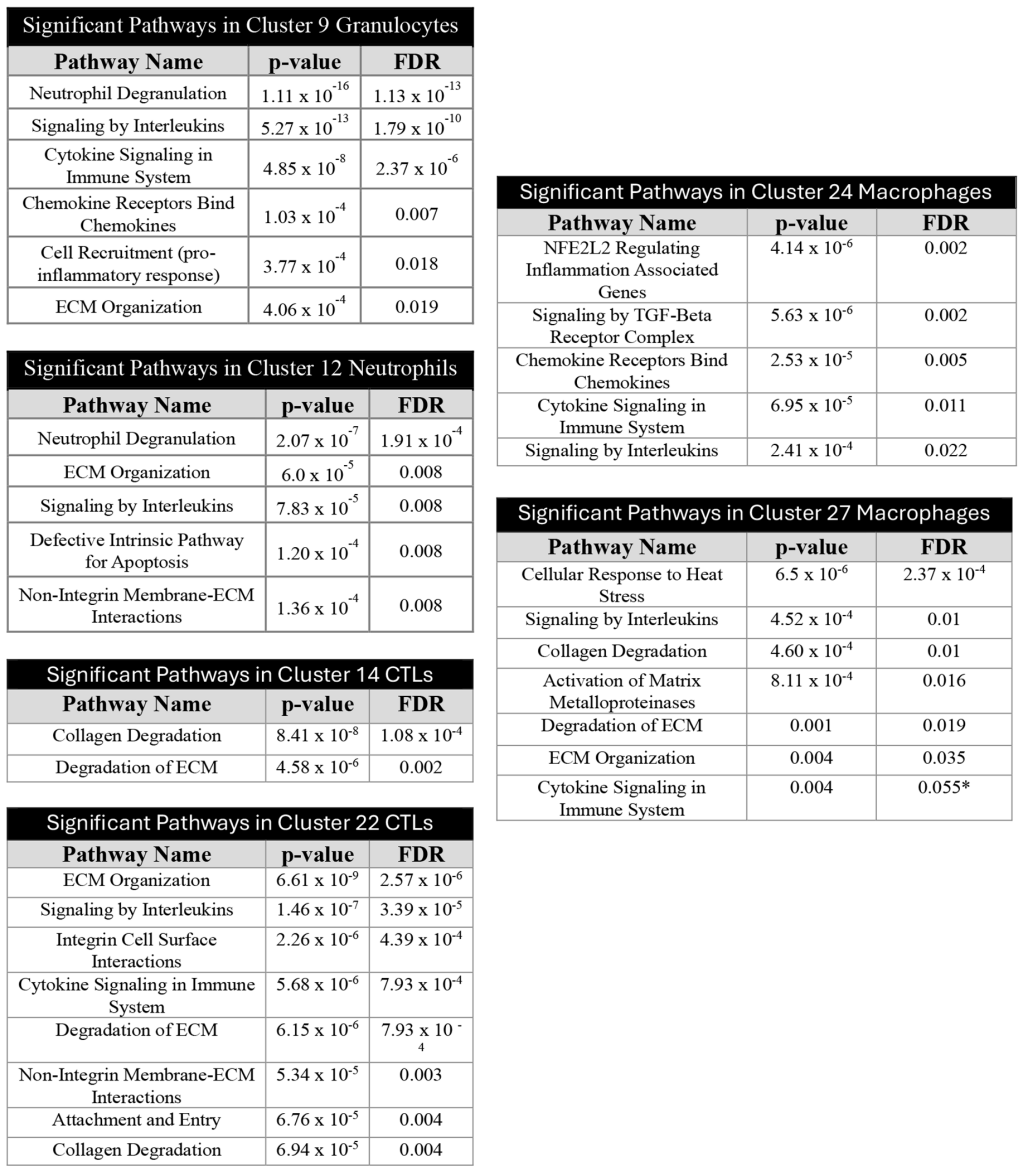
Altered gene expression for ECM organization, ECM degradation, and immune responses in immune cells. After identifying the uterine immune cells and DEGs in immune clusters, we performed pathway analyses using Reactome. Reactome performed over-representation analyses on DEGs in *Tsc2*-knockout mice identified which pathways are enriched in each cluster. The enriched pathways in each cluster are shown in tables with statistical results. Statistical analyses performed as described in Methods (see Refs. 68 and 69).

These transcriptomic data demonstrated enrichments of immune cell types as well as cytokine and chemokine receptor-ligands. To understand the potential effect of these changes on cell functions, we used Reactome [68,69] to perform pathway analyses on all DEGs in each immune cell cluster (Figure 3). Multiple pathways showed enrichment of genes in the *Tsc2*-knockout uteri in multiple clusters; these included extracellular matrix (ECM) organization and degradation (9, 12, 22, 27), collagen degradation (14, 22), signaling by cytokines/interleukins, attachment and entry, and cell recruitment. These data suggest functional changes in immune cells that affect tissue structure maintenance, infiltration, and cytokine-driven activation in response to an inflammatory tissue environment.

### Inflammatory cytokines are concentrated in the *Tsc2^fl/fl^PR^Cre^* mice sera

**Figure 4.**
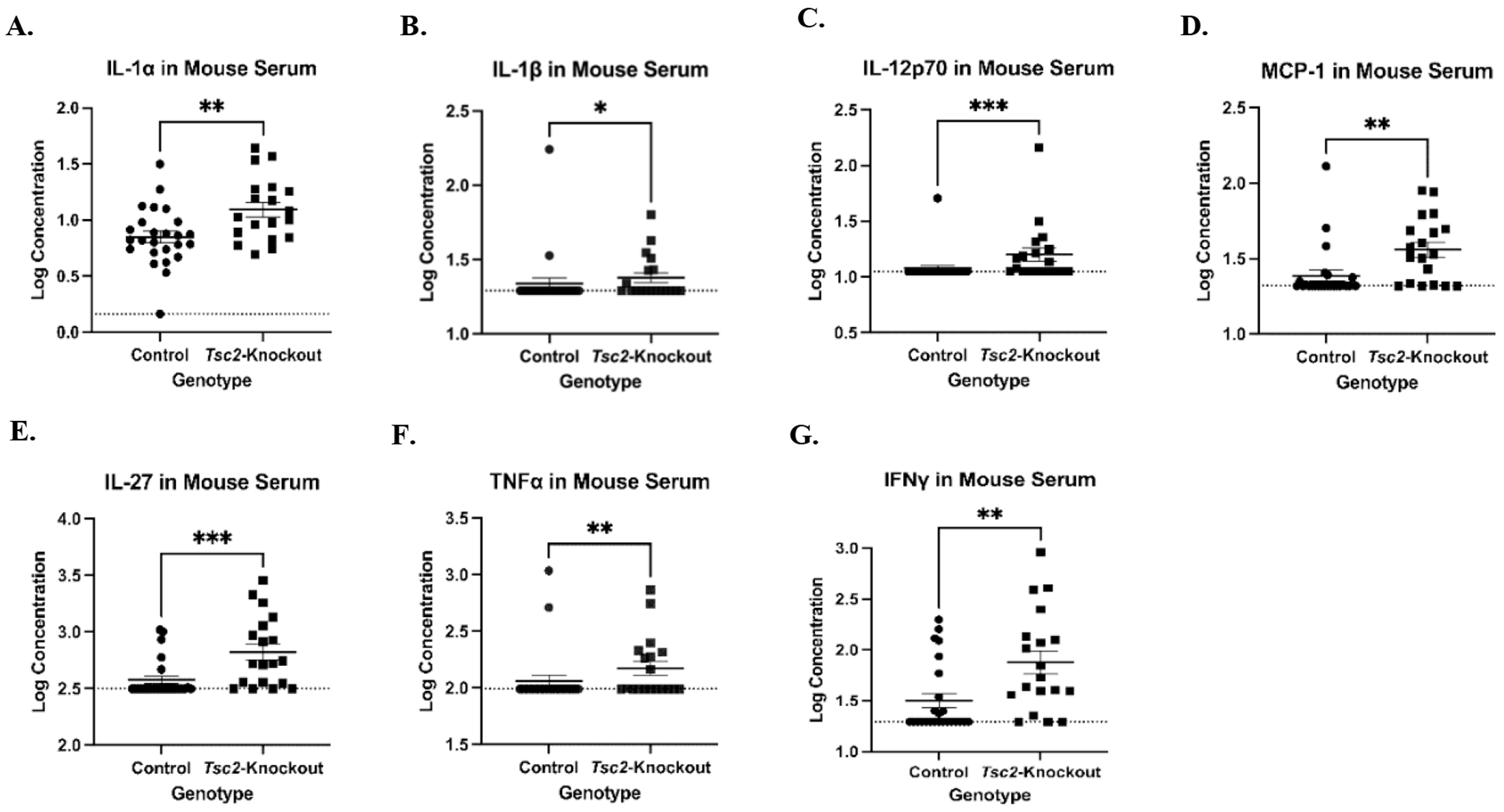
Inflammatory cytokine profiling of *Tsc2*-knockout mice demonstrates a pro-inflammatory milieu that would influence immune cell function. Serum was collected from control and *Tsc2*-knockout mice aged between 14-32 weeks old. A standard 13-cytokines inflammatory LEGENDPlex assay was performed to profile the cytokines in these mice as described in Methods. **A.-G.** Cytokine expression is significantly increased in the sera of *Tsc2*-knockout mice. For the groups, n=25 controls and 19 *Tsc2*-knockout, one assay performed, 3 technical replicates per sample, data are log transformed, and dotted line indicates the Limit of Detection. Graphs are mean ± SEM and Mann-Whitney test at p=0.05 performed.

Based on bioinformatic analyses, we examined the overall inflammatory state of *Tsc2*-knockout mice to determine if transcriptional changes were associated with cytokine protein levels. Serum was collected and analyzed using an inflammatory LEGENDPlex assay. This bead-based ELISA allowed us to profile 13 different inflammatory cytokines per sample via flow cytometry. We noted 7 that were significantly higher in concentration in the serum of *Tsc2*-knockout mice (Figure 4): IL-1α (Figure 4A), IL-β (Figure 4B), IL-12 (Figure 4C), MCP-1/CCL-2 (Figure 4D), IL-27, (Figure 4E), TNFα (Figure 4F), and IFNγ (Figure 4G). These data suggest that increased concentrations of pro-inflammatory cytokines within the *Tsc2*-knockout mice likely originate from multiple cells to influence the number and function of the immune cells in the uterus.

### Decreased uterine NK cells in *Tsc2^fl/fl^PR^Cre^* tissue

**Figure 5.**
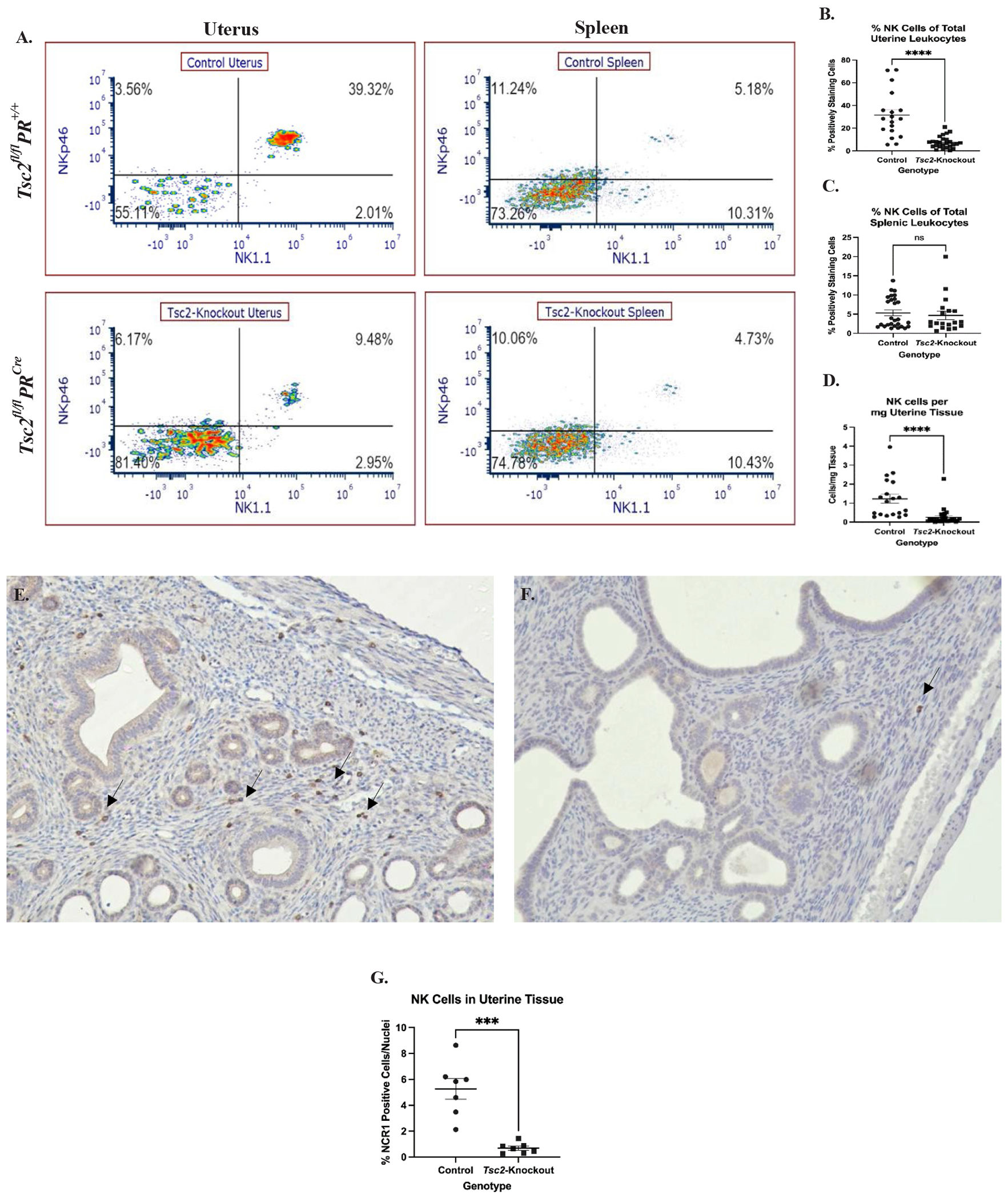
Decreased uNK cells in *Tsc2-*knockout mice uteri. Phenotyping was performed to assess the composition of the uterine cytotoxic lymphocytes. CD45^+^CD3^−^NK1.1^+^NKp46^+^ defined uNK cells as described in Methods. **A.** Representative flow cytometry plots demonstrating the difference in uNK cell incidence between control and *Tsc2*-knockout mice. Spleens were stained alongside as an internal control. **B-C.** Quantification of percent positively staining cells of splenic NK cells (**B.**) and uNK cells (**C.**) per total leukocytes. **D.** Normalization for differences in tissue mass digested to compare uNK cells abundance. Plots are cells per mg of tissue. **E.-G.** IHC was performed using an anti-NCR1 (NKp46) antibody to detect uNK cells. Representative images of control (**E.**) and *Tsc2-*knockout (**F.**) uteri are shown. Arrows denote positively staining cells within the tissue. **G.** Quantification of tissue uNK cells in tissue samples. For flow data, experiments were performed 10 times, n=19-27, and unpaired Student’s T test with p=0.05. Gating strategy is described in the methods. For IHC staining, experiments were performed 4 times, n=8 total for each group.

We further characterized the lymphocyte composition of the uterus to better understand the mouse model. Flow cytometry showed a marked decrease in the CD45^+^CD3^−^NK1.1^+^NKp46^+^ uNK populations from the *Tsc2*-knockout mice (Figure 5). Splenic lymphocytes were stained alongside uterine cells as an internal control. No differences in splenic NK cell composition between genotypes were detected (Figures 5A and 5C). Notably, the uNK cells are reduced in the *Tsc2*-knockout uteri (Figures 5A and 5B). Due to discrepancies in the amount of tissue digested for cell isolation, standardization based on the number of uNK cells per milligram of tissue was necessary; following this adjustment, a reduction in uNK cells in *Tsc2*-knockout mice was still observed (Figure 5D). To further support the flow cytometry results, IHC against NCR1/NKp46 was performed to visualize distribution of cells in the tissue. NCR1 positive cells were detected throughout the endometrial and myometrial layers of the control uteri (Figure 5E). Conversely, few NCR1 positive cells were found in *Tsc2*-knockout uterine tissue (Figure 5F). Quantification of the IHC demonstrates a decrease in uNK cells in *Tsc2*-knockout uteri, consistent with the flow cytometry data (Figure 5G). Therefore, our *Tsc2*-knockout uteri show a significant lack of uNK cells.

### uNK cell exhibit increased apoptosis

**Figure 6.**
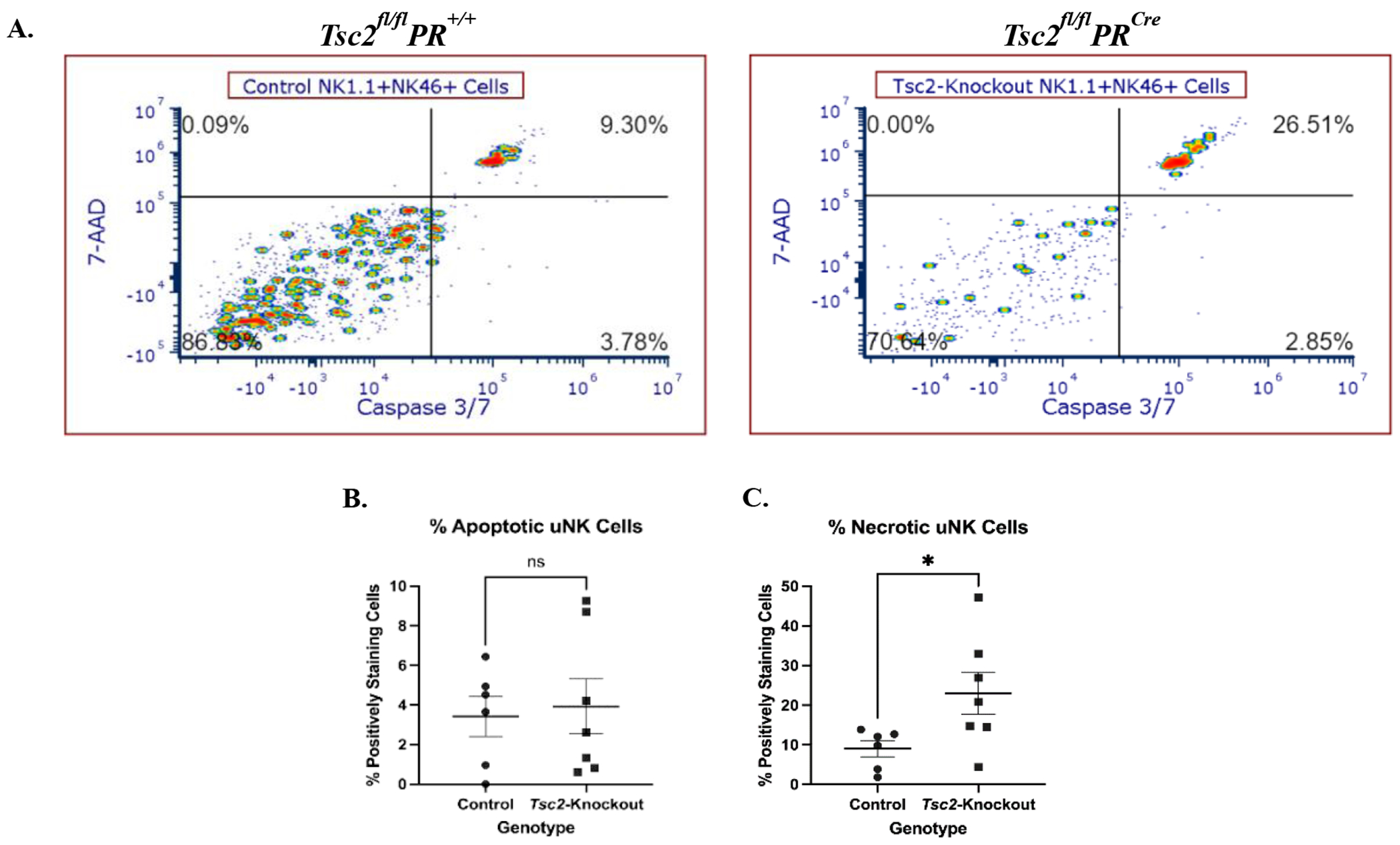
Increased uNK cell death results in a reduction in cell number in *Tsc2*-knockout mice. To investigate uNK apoptosis, a flow cytometry-based apoptosis assay employing a fluorescent Caspase-3/7 and 7-AAD staining was performed. Apoptosis was defined by Caspase-3/7 single staining, and necrosis, defined by Caspase-3/7 and 7-AAD dual staining, was measured on uNK cells from both genotypes. **A.** Representative flow cytometry plots of uNK cells shown. **B.** Quantification of the Caspase-3/7 single positive apoptotic cells. **C.** Quantification of the 7-AAD and Caspase-3/7 dual positive necrotic uNK cells. Spleens stained and analyzed alongside the uteri as an internal control comparison shown in Figure S5A. Experiments were performed 3 independent times, n=6-7 total, and Student’s unpaired t test performed at p=0.05. All graphs are mean ± SEM. Gating strategy is described in the methods section.

We hypothesized that the reduced uNK cell number results from stress induced by the hyperproliferative tissue environment, which ultimately leads to uNK cell death. Flow cytometry plots revealed bright populations in the upper right quadrant where the 7-AAD and Caspase-3/7 dual positive uNK cells reside (Figure 6A). There was no difference in the percentage of uNK cells in the early stages of apoptosis, defined by Caspase-3/7 single staining (Figure 6B). Conversely the fraction of necrotic uNK cells from *Tsc2*-knockout mice, distinguished by Caspase-3/7 and 7-AAD dual staining, was increased (Figure 6C). Splenic NK cells did not exhibit differences in apoptosis or necrosis between genotypes (Figure S5A). Therefore, the uNK cells from *Tsc2*-knockout mice are entering a Caspase-3/7 dependent apoptotic pathway at a higher frequency. This may provide a potential explanation for the decrease in cell number detected via flow cytometry and IHC experiments.

### Increased NKG2A expression on uNK cells

NK cell surface receptor expression is heterogenous. The balance of this surface receptor repertoire determines effector functions [22,25–28]. The activating receptor NKG2D recognizes and binds to stress-induced ligands (for example, MULT1/ULBP1, RAET1d/ULBP2) expressed on the surface of transformed cells. Activation of NK cells through this receptor is a strong and common pathway leading to target cell death [21,22]. In our previous study in humans, we found a lower percentage of PBMC NK cells expressing NKG2D, along with an overall decrease in receptor expression in LAM patients [56]. Based on these data, we hypothesized uNK cells exhibit similar alterations in NKG2D receptor expression, leading to changes in uNK cell functions and allowing for LAMCore cell proliferation in the uterus. We investigated the expression of NKG2D/CD314 on uNK cells. There was no difference in the percentage of uNK or splenic NK cells expressing NKG2D (data not shown). Thus, overstimulation of uNK cells through the NKG2D receptor pathway is likely not a contributing factor to increased uNK cell death and decreased tissue resident uNK cell numbers.

**Figure 7.**
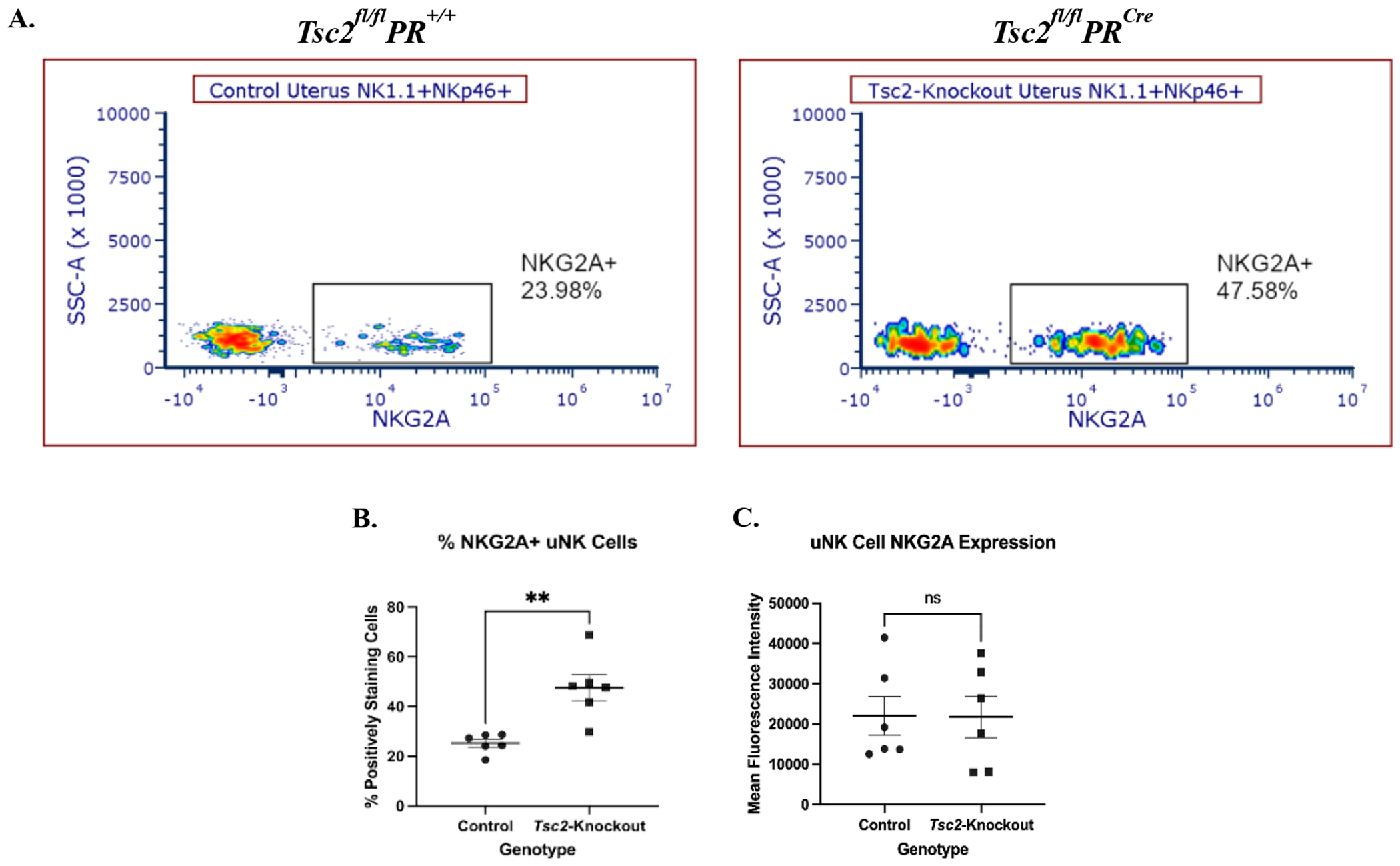
Increased NKG2A expression indicates dysfunctional uNK cells. The expression of the common inhibitory receptor NKG2A was measured via flow cytometry. **A.** Representative flow cytometry plots of NKG2A expression on CD45^+^CD3^−^NK1.1^+^NKp46^+^ NK cells from control and *Tsc2*-knockout uteri. **B.** Quantification of the percent of uNK cells expressing NKG2A and **C.** NKG2A expression by Mean Fluorescence Intensity (MFI). Splenic NK cells analyzed as an internal control shown in Figure S5B. Student’s unpaired t test performed at p=0.05, n=6-7. Graphs are Mean ± SEM.

Subsequently, we hypothesized the reduction in cell number is attributed to changes in expression of the inhibitory receptor NKG2A. This receptor, which dimerizes with CD94, identifies self-antigens to prevent unintended NK cell activation against healthy cells [19,20]. Expression of NKG2A often altered in disease states, resulting in NK cell dysfunction [77–80]. Flow cytometry analyses showed a distinctly positive population of NKG2A-positive uNK cells from uteri of both genotypes (Figure 7A). In *Tsc2*-knockout uteri, a higher percentage of uNK cells express the receptor (Figure 7B). However, the intensity of NKG2A expression between genotypes is not different (Figure 7C). As with previous data, the splenic NK cells remain unaffected in the fraction of cells expressing NKG2A or the amount of receptor expression (Figure S5B). From these data we hypothesize that more NKG2A expressing cells may represent a potential mechanism contributing to uNK cell dysfunction within the *Tsc2*-knockout uterine environment.

### uNK cells actively produce IFNγ

**Figure 8.**
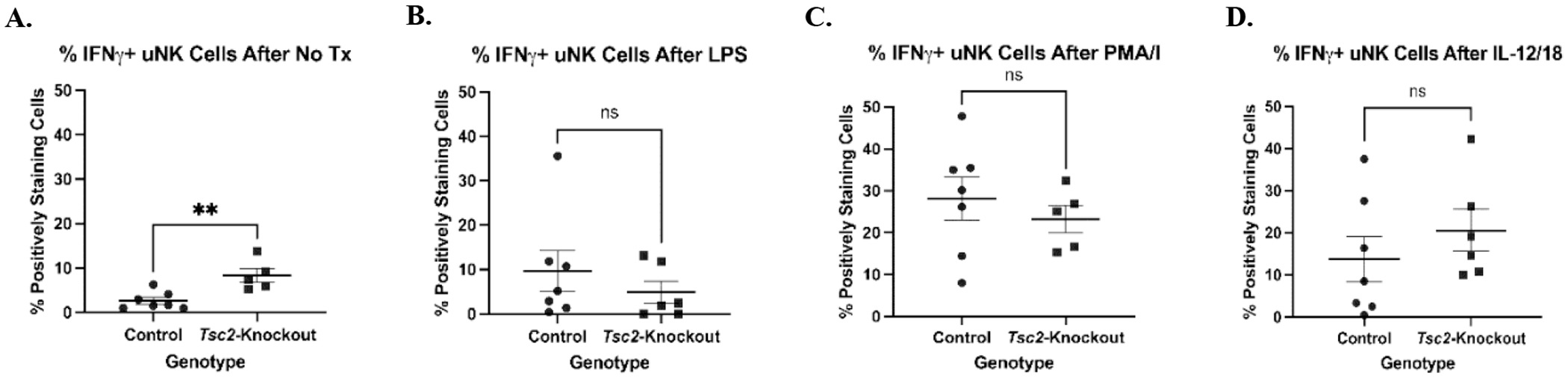
Increased uNK cell activity in *Tsc2-*knockout mice. Isolated lymphocytes were stimulated to assess functional responses as indicated by IFNγ production. Cells were pretreated with Brefeldin A to prevent cytokine release and allow for intracellular staining. **A.** IFNγ positive cells left in culture with maintenance doses of IL-2 and IL-15 only. **B.** IFNγ positive cells after maintenance cytokines in addition to LPS treatment. **C.** IFNγ positive cells after maintenance cytokines in addition to phorbol 12-myristate 13-acetate (PMA) and ionomycin (PMA/I) treatment. **D.** IFNγ positive cells after maintenance cytokines in addition to IL-12 and IL-18. Splenic lymphocytes were analyzed as an internal control shown in Figure S5C. All graphs show an n=5-7/genotype and Student’s T Test performed at p=0.05. All graphs are mean ± SEM.

The receptor expression alterations of NKG2A suggest changes in cell function. We hypothesized that uNK cells from *Tsc2*-knockout mice are hyporesponsive to external stimuli due to ongoing stimulation driven by stressful uterine environment developed during tissue hyperproliferation. We performed intracellular staining for IFNγ after various *in vitro* stimulations to test uNK cell function as these cells are the major producer of this pro-inflammatory cytokine [81–83]. uNK cells received one of the following treatments in combination with survival doses of IL-2 and IL-15: No treatment, LPS, PMA/I, or a combination of IL-12 and IL-18 (Figure 8). Cells were stained for IFNγ expression by flow cytometry. After stimulation with LPS and PMA/I, there were no differences in the uNK cells producing IFNγ (Figures 8B and 8C). IL-12/18 treatment also did not produce differential IFNγ production (Figure 8D). However, *Tsc2*-knockout uNK cells that did not receive additional stimulation produced more IFNγ at baseline (Figure 8A). Splenic NK cells did not demonstrate any differences in IFNγ production (Figure S5C). These data suggest the uNK cells from *Tsc2*-knockout uteri have a higher baseline activity when in an unstimulated environment. A lack of a significant response to known strong activators suggests a dysfunction in the uNK cell function in the *Tsc2*-knockout uterus.

### *In vivo* depletion of NK cells increases lung nodule formation

**Figure 9.**
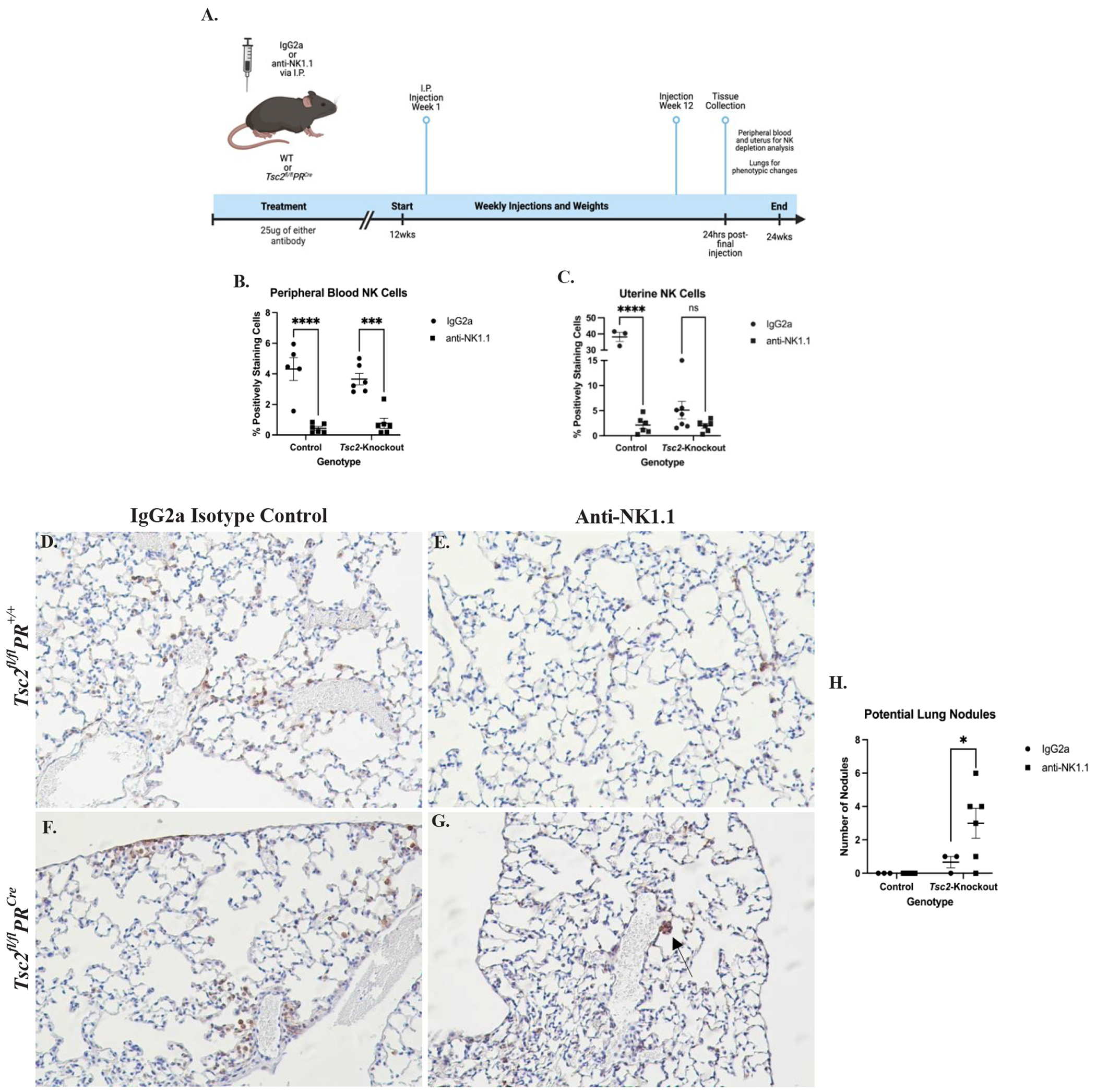
*In vivo* depletion of NK cells results in the development of pulmonary nodules at an earlier age. NK cell depletion was performed utilizing an anti-NK1.1 antibody. **A.** A visual schematic of the experimental design. Control and *Tsc2*-knockout mice were injected once/weekly with 25 μg of anti-NK1.1 or isotype control IgG2a antibody I.P. beginning at 12 weeks of age. After 12 weeks, mice were euthanized and tissues collected. Flow cytometry was performed on blood and isolated uterine cells. NK cells were defined as: CD45^+^CD3^−^NKp46^+^CD49b^+^. **B.** Peripheral blood stained for NK cells. **C.** Uterine lymphocytes isolated were collected and stained for NK cells. Panels **D.-G.** Representative staining of lungs from each cohort for pS6, a marker of cell proliferation through the mTORC1 pathway and LAMCore cells. **D.** and **E.** Control mice lungs that received isotype IgG2a control injections **(D.)** or anti-NK1.1 injections **(E.)**. **F.** and **G.** *Tsc2*-knockout mice lungs that received isotype IgG2a injections **(F.)** or anti-NK1.1 injections **(G.)**. **H.** Quantification of the number of nodule-like structures found in each individual mouse in each cohort. For all tissues and genotypes, n=3-6, experiment performed in two groups, Two-Way ANOVA performed at p=0.05. Graphs are mean ± SEM. Experimental graphic made using BioRender.com.

After *in vitro* studies, we performed *in vivo* studies to investigate the requirement for NK cells in the development of lung nodules. We hypothesized that the uNK cells cannot effectively control uterine overgrowth, allowing for the *Tsc2*-knockout LAMCore cells to proliferate and metastasize to the lungs. We selectively depleted NK cells *in vivo* using an anti-NK1.1 antibody injected intraperitoneally (I.P.) once a week for 12 weeks. Uterine and blood tissues were collected to assess NK cell depletion by flow cytometry, and lungs were inflation-fixed to use for IHC analyses. Animal weights were taken weekly prior to injection, and uteri weighed prior to digestion (data not shown) (Figure 9A). The effectiveness of the depletion was investigated by analyzing PBMC NK cells. Flow cytometry revealed that the composition of NK cells was not altered in mice receiving isotype control antibody treatment (IgG2a). When comparing the anti-NK1.1 group to the IgG2a isotype groups, NK cells were almost entirely depleted in the blood in both genotypes of treated mice (Figure 9B). Consistent with the PBMC NK cell data, control uNK cells were effectively depleted. Distinctly, the uNK cells from *Tsc2*-knockout mice were not significantly affected by the anti-NK1.1 antibody (Figure 9C). From these data, we hypothesize that the transformed uterine environment of the *Tsc2*-knockout mice prevents the anti-NK1.1 treatment from affecting the uNK cell populations.

During tissue collection, lungs were fixed to assess phenotypic changes. IHC staining for phospho-S6 ribosomal protein (pS6), which is a marker for proliferation through the mTORC1 pathway and is seen in LAMCore cells, was performed. No abnormal pS6-positive cells were noted within the lungs of control mice that received IgG2a (Figure 9D) or anti-NK1.1 antibodies (Figure 9E). In *Tsc2*-knockout mice that received IgG2a isotype control, the formation of nodule-like structures was not seen (Figure 9F). In contrast, in the lungs of *Tsc2*-knockout mice given anti-NK1.1 injections, smaller clusters of pS6-positive cells were observed throughout the tissue (Figure 9G). Control mice in each treatment group did not develop any nodule-like structures. *Tsc2*-knockout mice that received isotype control had no more than 1 nodule within the lungs. In contrast, the *Tsc2*-knockout mice that were depleted of NK cells developed significantly more nodule-like structures (Figure 9H). Therefore, depletion of circulating NK cells allows for increased formation of nodule-like structures in *Tsc2*-knockout mouse lungs implicating NK cells directly in the surveillance of nodule formation in LAM.

## Discussion

LAM is a rare cystic disease that destroys the lungs of women in their childbearing years [1–4]. Data convincingly suggests the uterus is the source of LAMCore cells present in the lung nodules [12–16,18]. Due to the delay in definitive LAM diagnosis, we used an immunocompetent mouse model that expresses a loss of *Tsc2* within the uterus to study early events in disease progression and establishment. This model mimics the origin of LAMCore cells by expressing the same gene loss in patients to enable the study of initial disease progression [57]. NK cells, which eradicate tumor cells, may be one of the lines of defense against proliferating LAMCore cells in the uterus. uNK cells are a significant percentage of immune cells and the most common of the lymphocytes in the non-pregnant uterus [45,71,72,84–86]. We hypothesized that uNK cells are dysfunctional and unable to control proliferating LAMCore cells prior to metastasis.

To study the early stages of disease, an immunocompetent mouse model was needed. For our studies, we utilized a uterine-specific *Tsc2*-knockout mouse model that maintains immunocompetency. The gene knockout is restricted to non-immune cells, which is consistent with the hypothesized myometrial origin of LAMCore cells in patients. The resulting phenotype is characterized by hyperproliferation of the endometrial and myometrial layers of the uterine tissue through the mTORC1 pathway [57,87]. Within the *Tsc2*-knockout mouse scRNAseq data, there is a large number of cells found in cluster 6 (Figure 2). This population is close to undetectable within the controls (Figure S3A). This suggests the population develops because of the gene knockout. The expression of well-established LAMCore markers *Gpnmb* and *Mlana* were both found within cluster 6 in *Tsc2*-knockout mice, while expression was absent in controls (Figures S3B and S3C). The expression of these markers in the cluster suggests a very promising population of cells that share gene expression profiles very similar to the LAMCore cells found in patients. Pathways enriched in these cells include chemokine signaling and focal adhesion through mTORC1 activation, the latter leading to downstream pro-survival pathways (Figure S4) [5]. The diseases associated with the cluster 6 cells include endometriosis, which is a disease of abnormal endometrial cell growth in and outside of the uterus [88], and cancer metastasis pathways. Interestingly, this cluster 6 differentially expressed multiple genes known to identify LAMCore cells in patient tissues while expression was not seen in control mice (Figure 2) [18]. From these data, we propose a potential population of cells in the *Tsc2*-knockout mice that mimic the LAMCore cells in patients. We hypothesize these cells develop as a result of the loss of *Tsc2*. However, more is required to fully characterize these cells before confidently referring to them as “LAMCore cells” that are potentially metastatic. It is possible these aberrant cells in our *Tsc2*-knockout uterus are producing pro-inflammatory cytokines to create systemic tissue inflammation (Figure 4), which has been described in other contexts [89,90]. Studies have shown mTORC1 activation in non-immune cells leads to the production of multiple pro-inflammatory cytokines to activate surrounding immune cells [91,92]. In a different *Tsc2*-knockout mouse model, IL-6 inhibition prohibited knockout cell proliferation [93]. The *Tsc2* deletion that occurs in our mouse model may augment the inflammation that already exists due to the cyclical building and breaking down of the uterine tissue during estrous. Rapid cell proliferation continues, and the inflammation is not resolved as it would in a normally cycling uterus. As cystic structures and necrotic tissue form during hypertrophy, uterine immune cells are activated in response. This leads to an inflammatory uterine environment to influence immune cell functions.

We performed scRNAseq on control and *Tsc2*-knockout uteri to gain insight into the changes that may have developed in the immune cells and structural cells, both of which influence the tumor microenvironment. Due to requirements for the assay, we sequenced all uterine cell types from fixed tissues to maximize cell numbers. In our scRNAseq data, 32 individual cell populations were identified in comparison to other published datasets (Figures 1A and 1B) [75,76]. We noted 11 individual immune cell clusters: B cells (11, 16, 32), DCs (25), macrophages (24, 27), neutrophils (12, 30), granulocytes (9), and CTLs (14, 22) (Figure 1F). In the immune cell populations, DCs were not noticeably different in cell number between genotypes (Figure 1J). More T and B cells were detected in control uteri while more neutrophils, granulocytes, and macrophages were detected in *Tsc2*-knockout mice (Figure 1G). Enriched N1 and N2 neutrophils in this mouse model have been previously reported [94,95]. Our granulocyte population expresses multiple genes of an N1 neutrophil alongside the neutrophil population expressing mainly N2 genes. The role of neutrophils in the tumor microenvironment is complex, with both pro- and anti-tumor functions arising in multiple subpopulations. Production of chemokines by tumor cells can recruit pro-tumor neutrophils into the tissue, furthering a metastatic environment. These neutrophils can be immunosuppressive against other immune cell types, contribute to ECM remodeling allowing metastasis, and drive the inflammatory environment. Anti-tumor neutrophils can also be found within the tumor environment. These cells effectively remove tumor cells themselves and through communication with other immune cells to bolster immune surveillance [135]. Given prior characterization of uterine neutrophils in *Tsc2*-knockout mice, we did not analyze them beyond the scRNAseq dataset. M1 and M2 macrophages have not been reported in the uteri of these mice. These will be the focus of future studies, as the altered functions of both subsets may influence the uterine immune microenvironment during the development and establishment of disease. Alterations of CTLs were also noted. Due to the unstudied nature of uNK cells in both this mouse model and LAM patients, our downstream studies focused on this cell population. From these data, our scRNAseq shows a difference in the composition of the immune environment in the *Tsc2*-knockout mice.

The uterine environment of *Tsc2*-knockout mice is inflammatory, potentially changing immune cell function. Therefore, we aimed to examine gene expressions for pro-inflammatory cytokines and receptors in the *Tsc2*-knockout uteri. In our single-cell data, *Il1a* and *Il1b* expressions are mainly expressed within neutrophils and granulocytes (Figure 1M). Accordingly, IL-1α and IL-1β are both more concentrated in the sera *Tsc2*-knockout mice (Figures 4A and 4B) and also use the same receptors [96]. Expression of the *Il1r1* is decreased in the macrophages from *Tsc2-*knockout mice (Figure 1N). Expression of *Il1r2* and *Il1rap* is not measured in the macrophage populations. This suggests that the macrophages found within the uterine tissue may not be activated by either IL-1 cytokine, but by another activating cytokine not present in the ELISA. The expression of *Il1r2* is mainly expressed in neutrophils in both genotypes. *Il1rap* expression shifts from mainly neutrophils in the controls to a more even expression between the neutrophils and granulocyte populations in the *Tsc2*-knockout mice (Figure 1N). Published data suggests that neutrophil recruitment occurs indirectly through activation with the IL-1 cytokines by driving expression of ligands for CXCR2. This activation can lead to neutrophil recruitment to areas of inflammation [97]. CXCR2 is promiscuous, as there are multiple ligands that bind to this receptor, including CXCL5 and CXCL2 [98]. Expression of *Cxcl2* is differentially expressed in multiple immune populations (Figure 1O). Previously published data by Taya *et al.* [95] determined enrichment of neutrophils in the *Tsc2*-knockout uterus by flow cytometry and bulk RNA sequencing. They report that myeloid-derived suppressor cells produce neutrophil elastase within the uterine tissue to promote neutrophil infiltration into the tissues. This trafficking is partially attributed to estrogen and *Cxcl5* and *Cxcr2* [94,95]. Our data supports their hypothesis of neutrophil and granulocyte infiltration into the tissue using the CXCR2 receptor-ligand axis. We show *Cxcl2* expression by multiple cell types, such as macrophages which have been reported to produce CXCL2 to recruit neutrophils (Figure 1O) [99,100]. In addition, Taya *et al.* demonstrate *Cxcl5* expression drives alterations in polymorphonuclear (PMN) cell composition [95]. Granulocytes in our data show increased *Cxcl2*, suggesting resident cell production to further recruit more granulocytes and neutrophils to the uterus. In addition to macrophages, activated NK and CD8^+^ T cells have been shown to produce CXCL2 in states of inflammation and activation [101,102]. Our single cell data shows increased *Cxcl2* expression by cluster 14, which contains both these cell types. Therefore, we hypothesize inflammation by IL-1 within the tissue drives CXCL2 production by multiple immune cell types to further recruit neutrophils and granulocytes to the tissue.

In our scRNAseq data, more macrophages were found within the tissue. Therefore, we hypothesized there is production of chemoattractant proteins for these cells. Our ELISA assay measured an increased concentration of MCP-1 within the sera of aged *Tsc2*-knockout mice, recapitulating expression in LAM patients [103]. MCP-1 is a chemoattractant produced by multiple cell types that can attract multiple immune cells, such as NK cells [104,105], but is most studied in the context of monocyte and macrophage populations. MCP-1 production is stimulated by inflammatory cytokines such as IL-1, TNFα, and IFNγ [106–111]. Our data supports tissue infiltration and differentiation of circulating monocytes into macrophages, as there is not a strongly distinguished population of monocytes within the *Tsc2*-knockout uteri. There are distinct macrophage populations in the tissue, based on expression of macrophage markers such as *Adgre1*, *Siglec1*, and *Cd163* (Figure S1). Expression of *Cd68* indicates macrophage activation. The macrophages in the *Tsc2*-knockout uteri show enriched expression of MCP-1 (Figure 1M). Trafficking and activation of circulating monocytes via MCP-1 and differentiation into macrophages in the tissue likely contributes to the enrichment of these cells in the *Tsc2*-knockout mice. Future studies will investigate the phenotype and function of these cells.

In our immune cells, DEGs for cell trafficking and tissue infiltration pathways were significantly enriched (Figure 3). These pathways were noted in macrophages, neutrophils, granulocytes, and cluster 22 CTLs, but not cluster 14 CTLs in *Tsc2*-knockout mice. This is of note, as data suggests uNK cell populations are supplied by circulating NK cells [41–43]. Increased expression of chemoattractant genes such as *Cxcl2*, *Cxcl6*, and *Cxcr6* is shown on uNK cells, suggesting the cells have migrated successfully into the uterus. While macrophages, neutrophils, and granulocytes are enriched, uNK cells are decreased in the scRNAseq on *Tsc2*-knockout uterus (Figure 1K). Our subsequent studies focused on the uNK cells due to their role in tumor surveillance and reported alterations of PBMC NK cells in patients. The discrepancy of uNK cells was also shown using flow cytometry and IHC (Figure 5). Strikingly, our studies that utilized splenic NK cells showed no changes in the population. This suggests defects in the infiltration of NK cells into the uterine tissue despite chemoattractant production and implies an unknown mechanism inhibiting NK cell trafficking. This results in a lack of sufficient uNK cell numbers, measured in multiple experiments, to adequately survey the uterus.

Our analyses clearly showed a decrease in the number of uNK cells isolated from the *Tsc2*-knockout mice (Figure 5). This was unexpected, as control uNK cells remained consistent outside of the expected fluctuations in cell numbers that are associated with the estrus cycle [112,113]. While trafficking may explain one method of reduced numbers, we hypothesized the stressful tissue environment that develops as a result of unregulated tissue proliferation due to the loss of *Tsc2* may drive uNK cell death. From this, we assessed apoptosis as an explanation of decreased uNK cell numbers. A higher percentage of uNK cells from *Tsc2*-knockout mice were in the later stages of apoptosis (Figure 6). We hypothesize uNK cells begin at normal numbers prior to reproductive maturity in the *Tsc2*-knockout mice. When *Tsc2* is knocked out due to *PR^Cre^* activation at reproductive maturity, cellular hyperproliferation initiates through the mTORC1 pathway. This results in the inability of the uNK cells to maintain pace with the rapidly developing tissue stress. The overstimulation of NK cells through constant activating receptor engagement has been reported in the context of other diseases, and the increased NK cell apoptosis we observed is consistent with this, leading to NK cell death or functional insufficiency [77–80,114,115]. Therefore, a fraction of the tissue-resident uNK cell population dies in response to continual stimulation. Cell numbers are not replenished due to issues with circulating NK cell trafficking into the tissue. This leads to a lower number of functionally insufficient tissue-resident uNK cells to respond to LAMCore cells. From these data, we hypothesize the *Tsc2*-knockout uteri have developed an inflammatory environment specifically affecting uNK cells. This environment prevents circulating NK cell infiltration and tissue residency, uNK cell function, and ultimately cell number through infiltration dysfunction and apoptosis.

Immune cell populations utilize pro-inflammatory cytokines to activate additional cells in the surrounding environment. One of these cytokines is IL-12, which was significantly increased within the sera of aged *Tsc2*-knockout mice (Figure 4C). Multiple cell types reportedly produce the cytokine [116–120]. Expression of *Il12a* is only detected in B cells in the control uteri (Figure 1M), while macrophages, neutrophils, granulocytes, and B cells express the cytokine in the *Tsc2*-knockout uteri. IL-12 is a known activator of multiple immune cells, including NK cells [83,121–123]. In the CTL populations, there is upregulated expression of both *Il12rb1* and *Il12rb2* (Figure 1N). These data suggest that the IL-12 measured in the serum may have functional effects on CTLs within the *Tsc2*-knockout uterus. Evidence has shown that cytokines can synergize together to elicit a stronger response in target cells [124,125]. This includes other members of the IL-12 family of cytokines, such as IL-27. This is a pleiotropic cytokine [126] that is concentrated in the sera of aged *Tsc2*-knockout mice (Figure 4E). In control mice, *IL27a* expression is almost exclusively shown in the DCs, while neutrophils, granulocytes, and macrophages are the main expressors in the *Tsc2*-knockout mice. Expression of the IL-27 receptor gene *Il27ra* is seen in the B and CTL clusters in both genotypes (Figure 1N). IL-27 has been reported to enhance NK cell function when combined with other activating cytokines IL-12, IL-18, and IL-15 [127,128]. Activated macrophages can produce IL-27 in response to other inflammatory signals [129,130]. It is possible that activation of macrophages, neutrophils, and granulocytes within the uterine tissue leads to production of IL-27 and IL-12. Dysregulation of uNK cells due to stimulation with these cytokines may lead to apoptosis and lower uNK cell numbers.

It is well known that activation of the CTLs through the IL-12/ IL-12R axis leads to IFNγ production. The CTL populations are the only cells expressing *Ifng* (Figure 1M), suggesting these are the main producers of the cytokine. The ELISA data showed increased IFNγ in the *Tsc2*-knockout sera (Figure 4G). Data from human immature CD56^bright^CD16^dim^ NK cells suggests that IL-1β synergizes with IL-12 to enhance IFNγ production by the cells [131]. In our model, uNK cells produce more IFNγ without additional stimuli (Figure 8A). This may be attributed to uNK cell response to higher concentrations of both IL-1β and IL-12 found in *Tsc2*-knockout mice. Both of the cytokines, along with IL-1α, may be attracting other immune cells and activating the NK cells within the uterus, leading to uNK cell dysfunction.

The effects of IFNγ are not restricted to CTLs. Expression of the IFNγ receptor genes *Ifngr1* and *Ifngr2* is detected within multiple immune cell types in both genotypes; *Ifngr1* expression is within the DC clusters and *Ifngr2* is found within the B, DC, macrophage, neutrophil, and granulocytes clusters (Figure 1N). IFNγ can trigger a response in non-lymphocytes such as neutrophils. Published data reveals that IFNγ activation of neutrophils leads to the production of Reactive Oxygen Species (ROS) and inflammatory cytokines such as TNFα [132]. Gene expression for *Tnf* is almost exclusively found within the granulocytes of *Tsc2*-knockout mice (Figure 1M). TNFα was detected at a significant increase in the sera of the *Tsc2*-knockout mice (Figure 4F). This suggests that the granulocytes found in cluster 9 are the main producers of TNFα in the *Tsc2*-knockout mice in response to IFNγ and other cytokines. Macrophages can also respond to IFNγ leading to M1 polarization and development of a pro-inflammatory phenotype [74,133,134]. Activation with IFNγ has been reported to lead to macrophage TNFα production, however gene expression of *Tnf* is not found in the macrophage clusters in our scRNAseq. This suggests other pathways, such as production of MCP-1, lead to macrophage activation in the *Tsc2*-knockout uterine tissue. This produces more pro-inflammatory signals to further activate the immune cell populations, potentially resulting in a pro-inflammatory feedback loop.

NK cells regulate receptor expression in response to a stressful and inflammatory environment. NKG2D expression on PBMC NK cells was different in LAM patients compared to healthy controls [56]. We hypothesized that this feature would be the same in the uterus of our disease model. However, no changes in NKG2D expression on uNK cells were identified. We measured the expression of NKG2A as an alternative. NKG2A is a well-described inhibitory receptor found on the surface of NK cells that recognizes the non-classical MHC molecule Qa-1 in mice or HLA-E in humans [19,20]. NK cells upregulate NKG2A expression in response to constant stimulation in a tumor environment. From this, NKG2A can serve as a marker for NK cell transition to cellular dysfunction [77–80]. Increased NKG2A-expressing uNK cells in the uteri of *Tsc2*-knockout mice was detected (Figure 7). We hypothesize the remaining fraction of surviving uNK cells upregulate NKG2A and are dysfunctional. This prevents the uNK from effectively killing proliferating *Tsc2*-null LAMCore cells. Our stimulation assays support this, as response to known activators is no different in the *Tsc2*-knockout uNK cells. This suggests that the inflamed *Tsc2*-knockout uterine environment constantly stimulates the uNK cells, leading to a lack of response. This, in combination with increased inhibitory receptor expression, suggests dysregulation of normal NK effector function. Therefore, the uNK cells cannot adequately respond to another stimulation when necessary.

In our scRNAseq dataset, immune cells show enrichment for DEGs in pathways related to tissue structure. These pathways include ECM organization and degradation, collagen degradation, and matrix metalloproteinase (MMP) activation (Figure 3). These enriched pathways in macrophages, neutrophils, and granulocytes suggests the cells are producing proteins to break down tissue to aid in trafficking and infiltration of other immune cells into the tissue, demonstrated by higher numbers of these cells. The breakdown of tissue structure may not only allow for immune cell infiltration. The activated immune cells breaking down the tissue suggests an easier pathway for the rapidly proliferating LAMCore cells to escape from the tissue and into the lymphatics, as this is the hypothesized pathogenesis [2]. This data provides an additional facet to LAMCore cell escape from the uterine tissue.

The development of lung manifestations during depletion of NK cells suggests a central role of NK cells in regulating disease progression in LAM. Control uNK cells were successfully depleted, while the *Tsc2*-knockout uNK cells were not. This lack of depletion suggests changes to the *Tsc2*-knockout uterine immune environment that prevents the antibody from effectively targeting the uNK cells. PBMC NK cells were successfully depleted in both genotypes. PS6 IHC revealed the formation of small, nodule-like structures distributed throughout the lungs of anti-NK1.1 treated *Tsc2*-knockout mice. This effect was not seen in either genotype of mice that received the IgG2a isotype control injections. From these data, we hypothesize that circulating PBMC and pulmonary NK cells play a role in controlling LAMCore cell circulation and seeding within the lungs. Without NK cells, the LAMCore cells can migrate and settle within the lungs sooner than if the NK cells were present.

There are limitations to our studies. This mouse model used for LAM develops an aggressive uterine hypertrophy that is not present in human patients. This may drive changes to the uterine environment not seen in patients. Our flow data was performed with limited cell surface makers, potentially missing subpopulations. Future studies will expand on this aspect to investigate all immune subpopulations within the tissue. Our studies provide phenotypic and limited functional changes in the uNK cell population. Future studies focusing on the uNK cells will be performed, such as anti-NKG2A treatment to further examine potential cell dysfunction through this pathway. Our NK cell depletion study demonstrated a potential issue with targeting uNK cells in the *Tsc2*-knockout uterus. However, no mechanistic studies were performed to further support this idea. Future studies will focus on elucidating these mechanisms in the uterine immune microenvironment. The data presented provides a snapshot into NK cell dysfunction in our model. However, these cells may change over time. Future studies aim to evaluate changes over time as the mice age and develop the pulmonary nodule phenotype. Our data suggests NK cells play a role in controlling metastasis of aberrantly proliferating cells from the uterus to establish disease in the lungs. Potential dysfunction of uNK cells was presented. However, data are from mice at similar ages and the function may change over the course of aging the mice. Future studies will include longitudinal phenotyping and function over aging to measure how function may change with disease progression in our model. Lastly, this study focuses on the uterine immune environment; however, patient mortality is driven by pulmonary failure. Future studies will utilize this model to investigate the pulmonary immune environment, which remains understudied in LAM.

## Conclusions

Our data, taken together, clearly demonstrate that the tissue specific uterine immune microenvironment plays a critical role in the pulmonary seeding of LAMCore clusters. To date, the role of immune state in this early disease establishing biology has not been examined. We have shown by scRNAseq that the inflammatory environment is significantly altered, and neutrophils, granulocytes, and macrophages are enriched in *Tsc2*-knockout uteri. In addition, uNK cells are reduced in number and data suggests dysfunction. When NK cells were ablated, we observed a much earlier establishment of *Tsc2*-knockout nodules in the lungs of these mice. This is very suggestive that NK cells play an important role in the pre-clinical stage of disease onset. Additionally, at least in mice, the increased inflammation present in the uteri, and the increased tissue degradation present, due to neutrophils, granulocytes and macrophages, may also play a role in a permissive environment for *Tsc2*-knockout LAMCore cells to metastasize. Elaborating the role of NK cells in the context of LAM will lead to a further understanding of the initial mechanisms of disease establishment, progression, and pathogenesis. These data, which will require further work, will aid in the understanding and development of potential therapeutics targeting, such as NKG2A blockade, as new treatment modalities for LAM.

## Supplementary Material

JCI-25-227-Supplementary_File

## Data Availability

Flow cytometry data deposited into FlowRepository. Single-cell RNA sequencing data deposited in GEO.
