# Supplementary material for: Inflammatory, Functional, and Compositional Changes of the Uterine Immune Microenvironment in a Lymphangioleiomyomatosis Mouse Model
Source: J Cell Immunol. Author manuscript; Available in PMC 2025 Aug 29. (PMC12392152; doi:10.33696/immunology.7.227)
Supplement: JCI-25-227-Supplementary_File [file NIHMS2101039-supplement-JCI-25-227-Supplementary_File.pdf]

Stiene DS, Osterburg AR, Corsarie LB, Balzarini NR, Medvedovic M, Borchers MT, Inflammatory, Functional, and Compositional Changes of the Uterine Immune Microenvironment in a Lymphangioleiomyomatosis Mouse Model. J Cell Immunol. 2025;7(3):74-97.

Supplement Figure 1

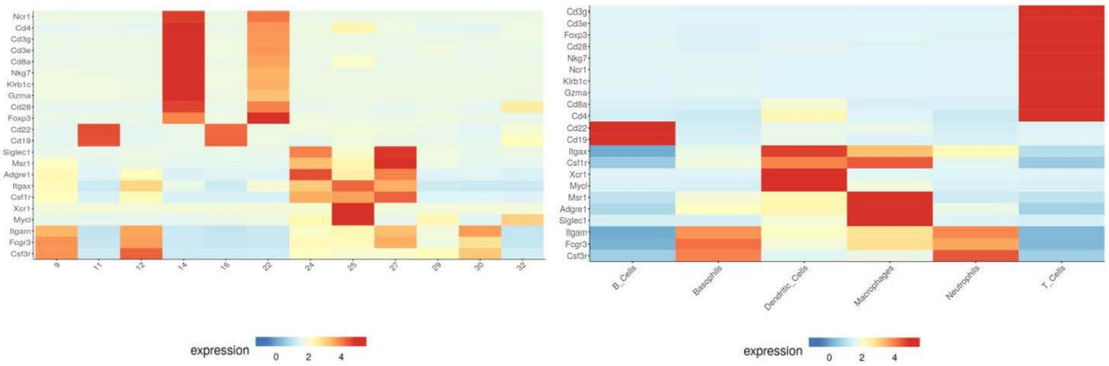

Supplemental Figure 1. Standard cell markers reveal multiple immune cell types and subtypes in uterine tissues. Using reported gene markers for various immune cell types in both genotypes of mice, all the clusters in the UMAP that express *Ptprc* populate with the cell markers. There are distinct B cells found in clusters 11 and 16 using *Cd19* and *Cd22*. T cells and NK cells are both found in clusters 14 and 22 based on expressions of *Cd3e/g, Foxp3, Cd28* or *Ncr1, Nkg7,* and *Gzma* but are grouped under the T cell labeling. Clusters 9, 12, and 30 show similar gene expression of neutrophil markers *Itgam, Fcgr3* and *Csf3r*, however, cluster 9 is labeled under basophils which have not been previously reported to reside in the uterus.

Therefore, we redesignated this cluster as granulocytes. Dendritic cells show similar gene expression to clusters 24 and 27, but the expressions of *Xcr1, Mycl,* and *Itgax* distinguish these cells in cluster 25. Clusters 24 and 27 express the macrophage markers *Siglec1, Msr1, Adgre1,* and *Csf1r* at varying levels, indicating these are related cells. These clusters are grouped under the macrophage label. Orange-red coloring indicates upregulation, blue coloring is down regulation, and yellow is no expression.

Supplement Figure 2

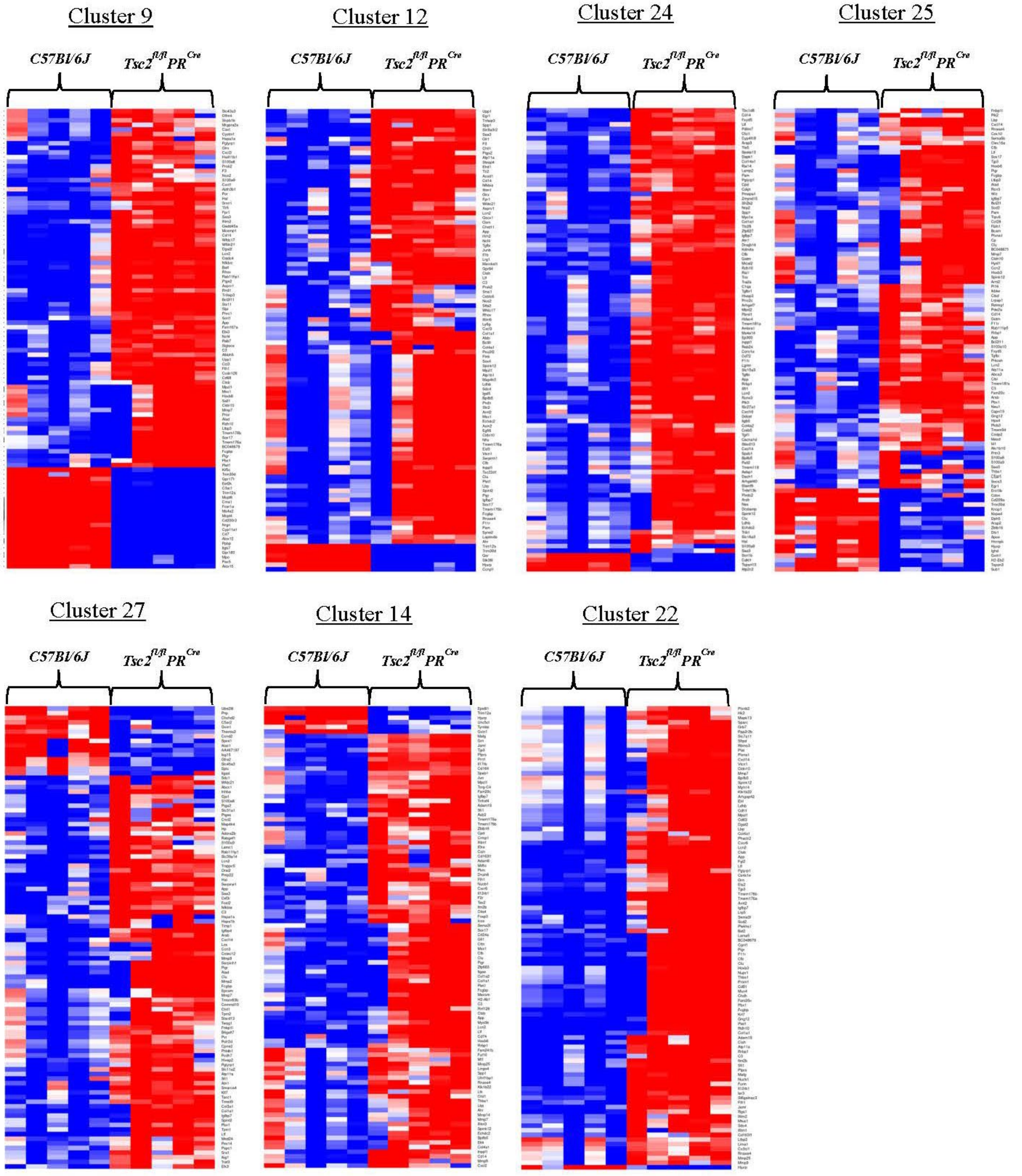

Supplement Figure 2. Cluster comparisons of neutrophil, granulocytes, macrophage, and CTL cell types reveal significant upregulation of multiple genes in *Tsc2*-knockout mice. After confirming the identity of immune cell populations, cluster analysis was performed to compare differential expression for changes in the *Tsc2*-knockout mice. Cluster 9 is granulocyte, cluster 12 is neutrophils, clusters 24 and 27 are macrophages, cluster 25 are dendritic cells, and clusters 14 and 22 are CTLs. Heatmaps display the top 100 DEGs in each cluster, 5 C57Bl/6J and 5 *Tsc2*-knockout mice.

Stiene DS, Osterburg AR, Corsarie LB, Balzarini NR, Medvedovic M, Borchers MT, Inflammatory, Functional, and Compositional Changes of the Uterine Immune Microenvironment in a Lymphangioleiomyomatosis Mouse Model. J Cell Immunol. 2025;7(3):74-97.

Supplement Figure 3

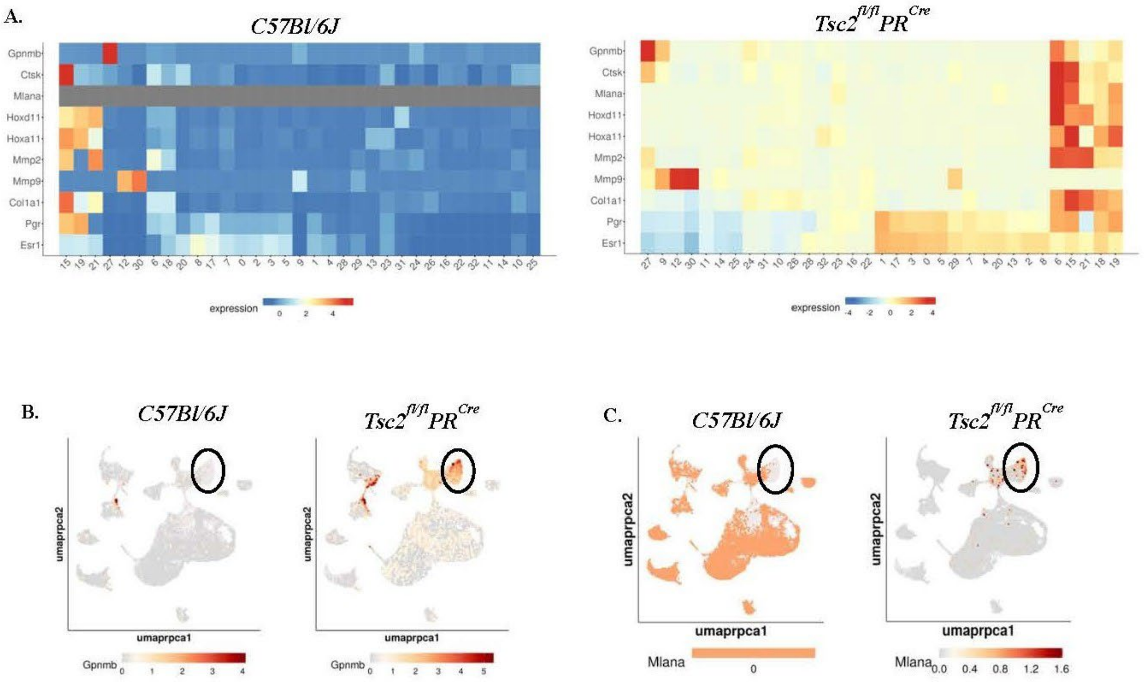

Supplement Figure 3. LAM cell markers are found in cluster 6. Control and *Tsc2*-knockout mouse clusters were analyzed for expression of multiple LAMCore markers (See Ref. 18). **A.** Heatmaps for LAMCore marker expressions in control and *Tsc2*-knockout mice. **B.** Expression of the specific LAMCore marker *Gpnmb* was investigated in control and *Tsc2*-knockout mice. Circles denote cluster 6. **C.** Expression of another specific LAMCore marker *Mlana* in control and *Tsc2*-knockout mice. Circles denote cluster 6. The orange color in the control UMAP indicates zero expression of the gene in all samples.

Stiene DS, Osterburg AR, Corsarie LB, Balzarini NR, Medvedovic M, Borchers MT, Inflammatory, Functional, and Compositional Changes of the Uterine Immune Microenvironment in a Lymphangioleiomyomatosis Mouse Model. J Cell Immunol. 2025;7(3):74-97.

Supplement Figure 4

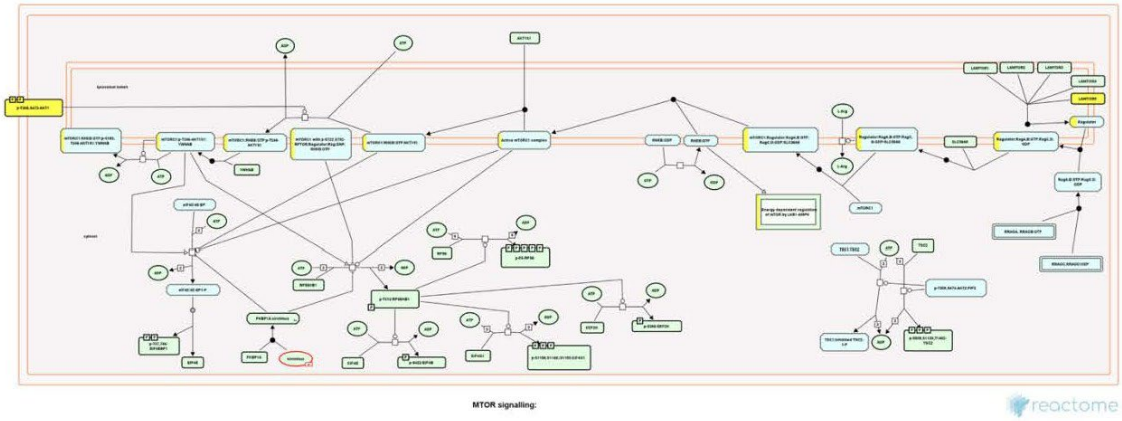

Supplement Figure 4. Enrichment of DEGs in the cluster 6 aberrant cells in *Tsc2*-knockout mice leads to mTORC1 pathway upregulation. DEGs from the comparison of cluster 6 between *Tsc2*-knockout and control mice were entered into the Reactome analysis feature. Within this cluster, multiple genes are overexpressed resulting in activation of the mTORC1 pathway. Boxes with yellow indicate locations where gene complexes are enriched in our gene list, leading to potential downstream functional changes. Image from Reactome (see Refs. 5, 44, 45), DOI: 10.3180/R-HSA-165159.1

Stiene DS, Osterburg AR, Corsarie LB, Balzarini NR, Medvedovic M, Borchers MT, Inflammatory, Functional, and Compositional Changes of the Uterine Immune Microenvironment in a Lymphangioleiomyomatosis Mouse Model. J Cell Immunol. 2025;7(3):74-97.

Supplement Figure 5

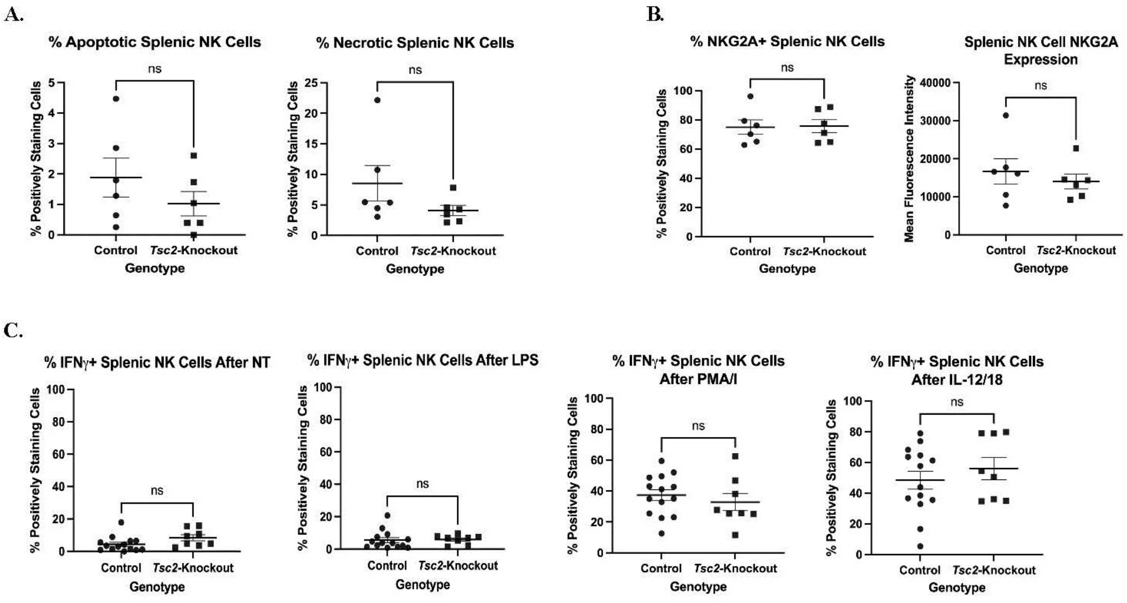

Supplement Figure 5. Analyses of splenic NK cells reveals no alterations in the *Tsc2*- knockout uterus. For all molecular biology experiments, splenic NK cells were analyzed alongside as an internal control. **A.** Apoptosis analyses of splenic NK cells in both genotypes. Quantification of apoptosis and necrosis is shown in the graphs. **B.** NKG2A expression on $CD45^{+}CD3^{-}$ $NK1.1^{+}NKp46^{+}$ splenic NK cells in both genotypes of mice. Quantification of the percent of NK cells expressing the receptor and intensity (MFI) is shown in the graphs. **C.** The percentage of splenic NK cells producing IFNγ after various treatments. All graphs and genotypes are a minimum of n=5-7 and Student's T Test performed at p=0.05. All graphs are mean ± SEM.
